# Symmetry Breaking of Human Pluripotent Stem Cells (hPSCs) in Micropattern Generates a Polarized Spinal Cord‐Like Organoid (pSCO) with Dorsoventral Organization

**DOI:** 10.1002/advs.202301787

**Published:** 2023-05-12

**Authors:** Kyubin Seo, Subin Cho, Hyogeun Shin, Aeri Shin, Ju‐Hyun Lee, June Hoan Kim, Boram Lee, Hwanseok Jang, Youngju Kim, Hyo Min Cho, Yongdoo Park, Hee Youn Kim, Taeseob Lee, Woong‐Yang Park, Yong Jun Kim, Esther Yang, Dongho Geum, Hyun Kim, Il‐Joo Cho, Sanghyuk Lee, Jae Ryun Ryu, Woong Sun

**Affiliations:** ^1^ Department of Anatomy Korea University College of Medicine Seoul 02841 Republic of Korea; ^2^ Department of Bio‐Information Science Ewha Womans University Seoul 03760 Republic of Korea; ^3^ Department of Biomedical Sciences College of Medicine Korea University Seoul Republic of Korea; ^4^ Geninus Inc. Seoul 05836 Republic of Korea; ^5^ Samsung Genome Institute Samsung Medical Center Sungkyunkwan University Seoul 06351 Republic of Korea; ^6^ Department of Pathology College of Medicine Kyung Hee University Seoul 02447 Republic of Korea; ^7^ Department of Life Science Ewha Womans University Seoul 03760 Republic of Korea

**Keywords:** dorsoventral organization, human pluripotent stem cells, microcontact printing, spinal cord organoid, symmetry breaking

## Abstract

Axis formation and related spatial patterning are initiated by symmetry breaking during development. A geometrically confined culture of human pluripotent stem cells (hPSCs) mimics symmetry breaking and cell patterning. Using this, polarized spinal cord organoids (pSCOs) with a self‐organized dorsoventral (DV) organization are generated. The application of caudalization signals promoted regionalized cell differentiation along the radial axis and protrusion morphogenesis in confined hPSC colonies. These detached colonies grew into extended spinal cord‐like organoids, which established self‐ordered DV patterning along the long axis through the spontaneous expression of polarized DV patterning morphogens. The proportions of dorsal/ventral domains in the pSCOs can be controlled by the changes in the initial size of micropatterns, which altered the ratio of center‐edge cells in 2D. In mature pSCOs, highly synchronized neural activity is separately detected in the dorsal and ventral side, indicating functional as well as structural patterning established in the organoids. This study provides a simple and precisely controllable method to generate spatially ordered organoids for the understanding of the biological principles of cell patterning and axis formation during neural development.

## Graphical abstract

Micropattern‐based symmetry breaking is utilized to generate polarized spinal cord organoids (pSCOs) with a self‐organized dorsoventral (DV) organization. The proportion of dorsal/ventral domains is controllable by the micropattern size and local circuits are separately developed on dorsal and ventral sides. This study suggests that functional and structural patterning is established in pSCOs, partially recapitulating the DV axis of human SC.

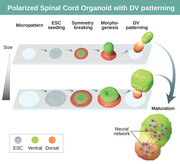

## 1 Introduction

Brain organoids are self‐assembled 3D cell aggregates derived from pluripotent stem cells (PSCs), which have now become an essential model system for human brain research as they recapitulate the cells and tissue structure of the brain, reflecting developmental trajectory. Much progress has been made in generating organoids of multiple brain regions^[^ ^1^, ^2^, ^3^, ^4^, ^5^, ^6^, ^7^, ^8^ ^]^ with diverse cell types.^[^ ^9^, ^10^, ^11^ ^]^ Bioengineered materials and fusion methods have been applied to develop more complex brain organoids.^[^ ^1^, ^5^, ^12^, ^13^, ^14^, ^15^, ^16^, ^17^, ^18^, ^19^ ^]^ However, one of the challenges in current organoid research is the establishment of the anterior‐posterior (AP), dorsoventral (DV), and mediolateral (ML) axes, which are crucial in the spatial organization of tissues and organs during embryo development. This fundamental patterning involves a break in the initial molecular and cellular symmetry, leading to the precise positioning of signaling centers. Signaling molecules released from these centers are distributed as a gradient, allowing cells to acquire discrete regional identities. Development of AP polarity has been reported in elongated gastruloids and 3D culture systems using blastocysts or epiblasts,^[^ ^20^, ^21^, ^22^, ^23^, ^24^ ^]^ and DV patterning is established in brain assembloids.^[^ ^13^, ^14^, ^15^, ^25^ ^]^ Recently, DV patterning in neural cysts which mimics neural tube development has been reported, although these studies focused mainly on early differentiation.^[^ ^25^, ^26^, ^27^ ^]^ To create a morphogen gradient in a highly controllable way, an engineered signaling center was introduced into one pole of forebrain organoids^[^ ^28^ ^]^ and a microfluidic approach has been utilized.^[^ ^29^, ^30^, ^31^ ^]^

Symmetry breaking and cell patterning using hPSCs have been relatively well studied in 2D cultures with geometric confinement. Simply confining cells to 2D micropatterns generates a spatially organized signaling environment in a controllable and reproducible manner, leading to regionalized cell fate patterning.^[^ ^32^, ^33^, ^34^, ^35^ ^]^ The spatial order of BMP4 signaling on micropattern is due to density‐dependent receptor relocalization and reaction‐diffusion of the noggin.^[^ ^36^ ^]^ Micropattern systems are also useful for studying the dynamics of morphogen signaling events during gastrulation.^[^ ^37^ ^]^ When micropatterned colonies stimulated with Wnt and Activin are grafted into chick embryos, they function as organizers, inducing secondary axis and neural tissue in the host.^[^ ^38^ ^]^ Additionally, micropattern technologies have been applied to recapitulate early human neurulation.^[^ ^39^, ^40^, ^41^ ^]^ All these studies provide good evidence that pre‐patterned geometric confinement can be a useful system for studying symmetry breaking and embryonic patterning using hPSCs.

Recently, several groups have reported the generation of spinal cord organoids,^[^ ^1^, ^2^, ^6^, ^7^ ^]^ but none of these organoids contained spatial patterning and functional arealization that are established in vivo in the spinal cord due to the absence of proper axes. In the present study, we took advantage of micropattern‐based symmetry breaking to generate an organized spinal cord organoid in which DV domains were spatially ordered, partially recapitulating the DV axis of the human spinal cord. The initial cell patterning induced by caudalization signals in 2D geometrically confined colonies autonomously developed into DV domains in caudalized 3D structures with unique protrusion morphogenesis. The characteristics of spatially organized organoids were analyzed using single‐cell RNA sequencing, immunostaining with spinal cord markers, and spatial transcriptomics, and were validated with DV axis perturbation experiments. Additionally, changing micropattern sizes enabled us to control the proportion of dorsal/ventral domains. In mature pSCOs, local circuits were separately developed on the dorsal and ventral sides, indicating that structural patterning led to functional patterning in polarized organoids. We suggest that geometrical confinement can be used to define the axis of 3D organoids, which is a flexible tool for mimicking early human spinal cord development in vitro. The structural and functional features derived from the DV‐like axis of pSCOs make our system more suitable for modeling developmental brain disorders and investigating the basic biology of human brain development.

## 2 Results

### 2.1 Spatial Cell Patterning in Geometrically Confined Human Embryonic Stem Cell (hESC) Colonies

**Figure 1:**
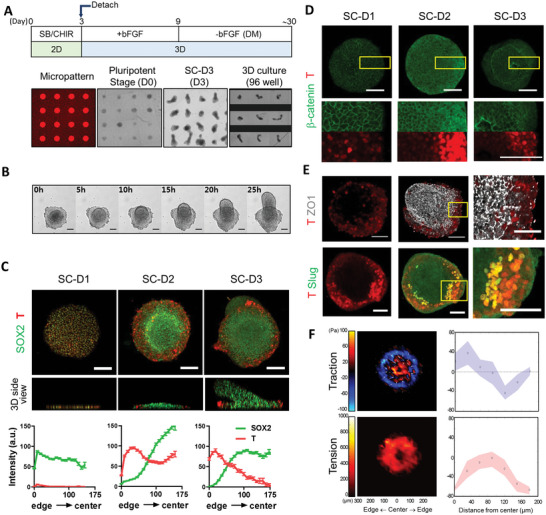
Spatial cell patterning and morphogenesis in geometrically confined hESC colonies. A) Schematic of the culture protocol. B) Bright‐field images of colony morphogenesis developing during the 3^rd^ day of SB/Chir treatment. C) Immunofluorescence analysis of SOX2 and T expressions. Quantification of fluorescent intensities at each position shown as mean ± SEM (*n* = total 7503 data points from nine images at SC‐D1, total 15 887 data points from 15 images at SC‐D2, total 15 085 data points from 11 images). D) Localization of GFP‐*β*‐catenin in micropatterned colonies of *β*‐catenin‐EGFP hiPSC cells during SB/Chir treatment. High‐magnification images shown at the bottom rows correspond to the insets. E) Loss of tight junction and Slug expression toward edges in micropatterned colonies treated with SB/Chir at day 3. High‐magnification images correspond to inset. F) A color‐coded map of radial coordinated cellular traction force with traction direction (white arrows) and tension at day 1 of SB/Chir treatment and quantification. The mean (black circle) and mid‐quartile (blue and red) obtained along the radial lines from the center to the edge of the cell colonies are shown. Confocal images were taken in 1‐ or 5‐µm steps along the *z*‐axis after fixation and immunostaining with the indicated antibodies and stacked using Z‐stack maximum projection. All images are representative examples from at least three independent experiments. Scale bar, 100 µm.

To generate polarized caudal 3D structures, we first induced 2D spatial patterning of caudal neural cells using geometrically confined hESC colonies following caudal neural induction protocols.^[^ ^6^, ^40^, ^42^ ^]^ Microcontact printing was utilized to generate micropatterns of matrigel‐coated adhesive circles with 350‐µm diameter on glass coverslips (**Figure** **1**A). Treatment with SB431542 (SB) 10 µM and CHIR99021 (Chir) 3 µM led to a marked protrusion of the central part of the micropatterned hESC colony, morphologically similar to “sprouting up” (Figure 1B, and Movie S1, Supporting Information). We found that the initial cell density on the micropattern at the beginning of neural induction is important in determining the occurrence of protrusion. When the micropattern was entirely filled with hESCs, center protrusion was rarely formed: they rather formed a dome‐like shape. In contrast, when the micropattern was less confluent, ring or bell‐shaped protrusions were more likely to form, and in this condition, more cells tended to form longer protrusions (Figure S1A, Supporting Information). In each experimental batch, we consistently observed ring or bell shapes resulting from sprouting morphogenesis with a yield of over 95%. The emergence of the neuromesodermal cells characterized by the co‐expression of SOX2 and T was evident on day 1 of SB/Chir treatment (SC‐D1), which was similarly seen in the SB/Chir‐treated hESCs under standard culture conditions (Figure 1C and Figure S1B, Supporting Information). At SC‐D2, the regional segregation on micropatterned colonies was evident; high expression levels of SOX2 were observed in most of the cells at colony centers, while T‐positive cells with low SOX2 levels progressively emerged at the edge of colonies (Figure 1C and Movie S2, Supporting Information). This segregation coincided with gradual cell accumulation at the colony center (0 h at Figure 1B and Movie S1, Supporting Information). At SC‐D3, “center” and “edge” cells were more clearly segregated and noticeable protrusions at colony centers grew upward over time.

It appeared that the preferential induction of T at the edge of the colony was associated with the strong Wnt activation at the edge. When Wnt activation by Chir was monitored by the cytosolic dispersion of *β*‐catenin, notably the colony edge that expressed high levels of T exhibited the *β*‐catenin accumulation selectively at the cytosol (Figure 1D). The resulting “edge” T‐positive cells lost tight junctions and expressed Slug, a marker for epithelial‐mesenchymal (EMT) transition^[^ ^43^ ^]^(Figure 1E). On the other hand, SOX2‐positive cells of the emergent protrusions at colony centers formed NCAD‐positive neural rosettes (Figure S1C, Supporting Information). All these data indicate that spatial differentiation patterning might occur through the interplay between the self‐organizing activity of the hESCs and cues from the geometric boundaries.

When Rac GTPase inhibitor (NSC23766) was applied to the micropatterned culture, both cell patterning and the center protrusion did not occur (Figure S1D, Supporting Information), raising a question of how these two events are related. Some colonies with higher initial cell densities failed to exhibit typical protrusion morphogenesis, but spatial patterning of SOX2/T expression was in most cases observed, indicating that geometric confinements faithfully triggered spatial cell patterning, while center protrusion morphogenesis requires the emergence of appropriate physical forces made by cell cluster with optimal density triggering the morphogenesis. Accordingly, we found that differential cellular physical force between the colony center and edge cells during SB/Chir treatment could contribute to center protrusion (Figure 1F). The traction map of the colony showed that cells at the edge developed inward pulling forces, indicating that cells tended to migrate out of the colony. Additionally, the tension map showed that high tension developed in the middle of the colony where cells started to aggregate and polarize. Based on these cellular force maps, it was clear that cells at the colony edge and center showed differential force development and distribution.

### 2.2 Development of 3D Structures from Micropatterned Colonies

**Figure 2:**
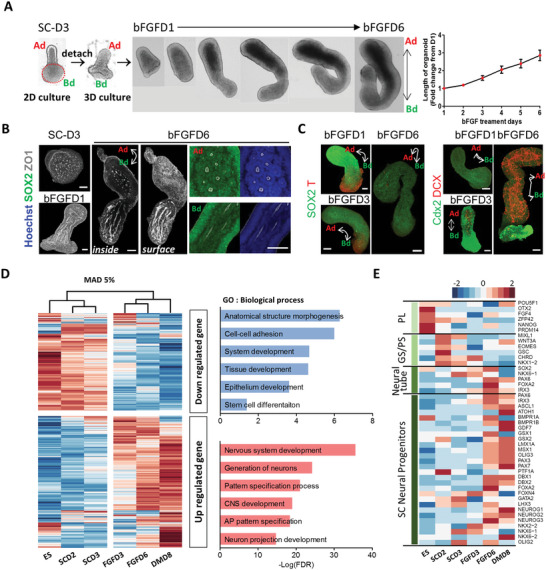
Characterization of polarized cell patterning and temporal gene expression profiles in 3D structures derived from 2D micropatterned colonies. A) Growth of 3D structures detached from 2D micropatterned colonies in the presence of bFGF for 6 days. The upper protrusive part was labeled as Ad (apical‐derived part), and the part attached to the micropatterned substrate was labeled as Bd (base‐derived part). Red dotted circle indicates the initially micropatterned area. Quantification of length of organoids during FGF treatment shown as mean ± SEM (*n* = 9 at each day). B) Immunofluorescence analysis of an attached colony (SC‐D3) and 3D structures (bFGFD1 and D6). White double arrowhead labeled as Ad and Bd indicates the long axis of organoids. Merged images of SOX2/ZO1 and Hoechst/ZO1 with high‐resolution show the rosette structures. C) Immunofluorescence analysis of indicated proteins in the 3D structures at the indicated time. D) Cluster analysis of microarray datasets using time‐pooled 2D colonies and 3D structures. The top 5% of most variable genes across samples were determined by the mean absolute deviation (MAD) function and clustered. The heatmap shows expression profiles of top 5% of most variable genes over time with functional annotation. E) Heatmap of gene expression associated with development over time. Well‐known marker genes for pluripotency (PL), gastrulation, primitive streak (GS/PS), neural tube, and spinal cord (SC) progenitors were examined at each stage. Confocal images were taken in 1‐ or 5‐µm steps along the z‐axis after fixation and immunostaining with the indicated antibodies and stacked using Z‐stack maximum projection. All images are representative examples from at least three independent experiments. Scale bar, 100 µm.

At SC‐D3, we gently lifted differentiated colonies with a center protrusion from the coverslips without disrupting their overall structures using the non‐enzyme‐based depolymerizing solution. Detached colonies from the substrate were composed of two parts: base (disk‐shaped bottom)‐derived (Bd) part and apical (center protrusion)‐derived (Ad) part (**Figure** **2**A). Each colony was transferred to an ultralow attachment 96‐well plate and cultured continuously in the presence of basic fibroblast growth factor (bFGF), which is required for induction of neural progenitor cells for additional 6 days (bFGFD1–6) (Figure 2A). While it has been reported that FGF signaling plays a role in regionalization along the AP axis,^[^ ^44^, ^45^, ^46^, ^47^ ^]^ bFGF2 did not affect further posteriorization in this 3D structure (Figure S2A, Supporting Information). The 3D structures underwent gradual reorganization, especially at the Bd part (Movie S3, Supporting Information). The entire 3D structure took the shape of peanuts and was further extended with a few bendings along their long axes in the presence of bFGF. The Ad part with densely packed cells was distinguishable from the Bd parts with a rather brighter appearance, which originated from the disk‐shaped bottom.

Two distinct types of internal cell arrangements were developed in each part: The Ad part cells were mostly organized to follicle‐like structures, while the Bd part cells preferentially formed tube‐like structures (Figure 2B and Movie S4, Supporting Information). The entire cells forming these 3D structures were SOX2‐positive, and T‐positive cells were confined to the Bd part and eventually disappeared (Figure 2C). The end domain of Bd parts containing T‐positive cells expressed cdx2, which is a member of the caudal‐related homeobox transcription factor.^[^ ^2^, ^48^, ^49^, ^50^ ^]^ Because we failed to observe any mesoderm lineage cells at this stage, it appeared that these T‐positive provisional mesodermal cells fail to maintain in our culture condition. DCX‐positive neuroblasts emerged at bFGFD3 and continuously increased (Figure 2C). This set of observations indicated that cells of micropattern‐derived 3D structures were differentiated into at least two morphologically distinct neural lineages during bFGF treatment. After 6 days of bFGF treatment, 3D structures were cultured without bFGF for further differentiation into neurons, and we referred to this stage of growing without bFGF as the DM (differentiation medium) stage.

Microarray analysis of temporal gene expression profiles revealed that our stepwise induction and differentiation protocol (Figure S2B, Supporting Information) produced early caudal neural stem/progenitor cells through the developmental stages of gastrulation and neurulation, which possibly matured into spinal cord cells. Gene transcripts associated with central nervous system development, AP pattern specification, and neuron‐related process were highly enriched in FGFD3, FGFD6, and DMD8 samples (Figure 2D). Specifically, genes associated with gastrulation and primitive streak were initially expressed, and then neural tube‐associated genes increased (Figure 2E). Marker genes for neural progenitors in the spinal cord started expressing at FGFD6. HOX gene clusters, up to HOX10, were gradually expressed, suggesting that our 3D structures held the caudal identity beginning from the hindbrain to the lumbar vertebra (Figure S2C, Supporting Information). Pathway and ontology analyses with Enrichr,^[^ ^51^, ^52^, ^53^ ^]^ showed that spinal cord‐related genes were enriched in the top 250 most variable gene sets, and interestingly, neural crest (NC)‐related genes were induced by our protocol (Figure S2D, Supporting Information). As NC cells arise from the neural plate border, it appears that the neural plate border was induced by our protocol aimed at directing caudal neural fate.^[^ ^48^, ^54^ ^]^ Based on our initial characterizations, we termed our 3D structures “polarized spinal cord organoids (pSCOs)” that were reminiscent of the early developmental stage of the embryonic spinal cord.

### 2.3 Single‐Cell Level Analyses of pSCO

**Figure 3:**
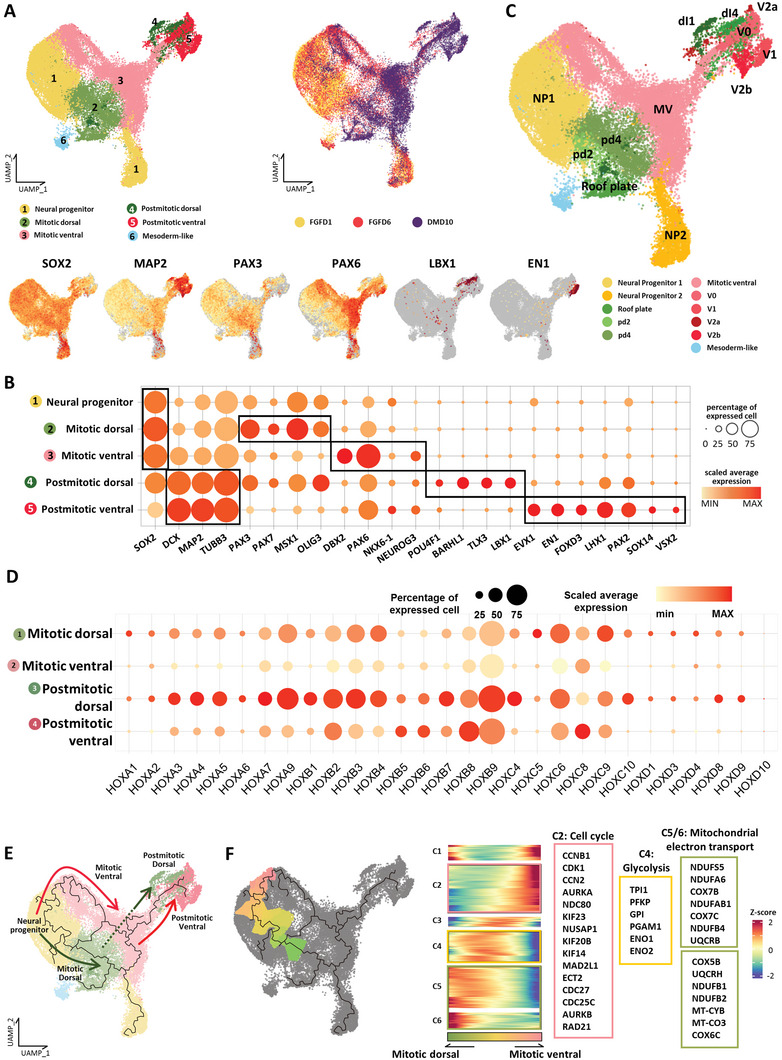
Molecular characterization of the organoid cell population via single‐cell RNA‐sequencing (scRNA‐seq). A) UMAP plot of integrated FGFD1, FGFD6, and DMD10 datasets. The six main cell clusters (left UMAP plot) and cells of different stages (right UMAP plot) are differently colored. UMAP plots at the bottom row show gene expression levels of representative signature genes for each cluster. B) Dot plot showing the expression of representative signature genes across five main clusters. The size of each circle indicates the percentage of cells in each cluster where the indicated gene was detected, and the color intensity reflects the scaled average expression level within each cluster. Genes in black boxes are representative signature genes in each cluster. C) Sub‐clustering of six main clusters in(A). Mitotic and postmitotic cells of organoids were differently colored according to expression profiles of dorsal and ventral spinal cord‐related genes. Domain‐unspecified cells were labeled with mitotic ventral. D) Dot plot showing the expression of HOX gene clusters across four main clusters. The size of each circle indicates the percentage of cells in each cluster where the indicated gene was detected, and the color intensity reflects the scaled average expression level within each cluster. E) Monocle‐based trajectory analysis of pSCOs. Each trajectory path is differently colored. F) Dorsal–ventral split in the neural progenitor cluster. Pseudotemporal gene expression profiles were hierarchically clustered. Genes related to indicated GO terms in each cluster are highlighted on the right side.

Single‐cell RNA‐seq was performed to understand the whole variety of cell types present in the pSCOs and their temporal changes unbiasedly. Specifically, three samples of FGFD1, FGFD6, and DMD10 were subjected to scRNA‐seq and 36 004 cells were further clustered and annotated into six main clusters: 1) Neural progenitor, 2) mitotic dorsal, 3) mitotic ventral, 4) postmitotic dorsal, 5) postmitotic ventral, and 6) mesoderm‐like clusters (**Figure** **3**A,B and Data S1, Supporting Information).^[^ ^55^ ^]^ The cell type composition showed a progressive development of pSCOs into neural cells over time. The portion of neural progenitor cells decreased in the order of FGFD1‐FGFD6‐DMD10 samples, while the postmitotic cells showed the opposite trend (Figure 3A and Figure S3A, Supporting Information). When each main cluster was sub‐clustered into the detailed neuronal cells types, we found that the organoids contained almost the full repertoire of spinal cord domains at both mitotic and postmitotic stages (Figure 3C, and Figure S3B and Data S2, Supporting Information). The mitotic dorsal cluster contained cells of the roof plate and dorsal domains of pd1‐pd6, and the mitotic ventral cluster had cells of p0‐p1‐p2‐pMN domains. The postmitotic dorsal and ventral clusters contained dI1‐dI3 and V0‐V1‐V2‐vMN domains, respectively. In addition, a gradual increase in HOX gene expression over time confirmed the caudal identity of our organoids (Figure S3C, Supporting Information). When comparing HOX gene expression across main cell clusters, there was no difference in their expression, even though most of the HOX genes were expressed more highly in dorsal clusters (Figure 3D). However, certain aspects of HOX gene expression pattern along the DV‐like axis in pSCOs were similar to the Hox patterns in mouse spinal cord.^[^ ^56^, ^57^ ^]^ For instance, HOXB4 and HOXD4 were slightly more expressed in the dorsal clusters, HOXB9 showed high expression in the dorsal part and HOXC8 was highly expressed in the ventral clusters. Previously published data from the developing mouse spinal cord^[^ ^58^ ^]^ were further used for independent validation of our cell type assignment (Figure S4A,B, Supporting Information). Additionally, we identified cell clusters related to neural crest cell (NCC) development, which was consistent with microarray data (Figure S2D, Supporting Information). Using a recent publication by Park et al.^[^ ^59^ ^]^ as a reference, we identified five distinct clusters that correspond to different stages of NCC development: neural plate border cells (PAX3+/Zic1+/MSX1+), roof plate cells (Lmx1a+/Msx1+/Msx2+/Wnt1+), Pre‐EMT NCCs (BMP6+/GDF7+), EMT NCCs (Dlx5+/Pdgfra+), and migratory NCCs (SOX10+/Foxd3+) (Figure S5A, Supporting Information). Furthermore, the RNA velocity analysis showed a trajectory of gene expression changes from neural plate border to roof plate and pre‐EMT state, although EMT and migration states were not included in the trajectory due to the limited number of cells in these states (Figure S5B, Supporting Information). The expression of HOX gene clusters in pre‐EMT and migratory NCCs suggests that NCCs in our organoids were more similar to vagal/trunk NCCs (Figure S5C, Supporting Information).

We also performed a trajectory analysis with Monocle3,^[^ ^60^ ^]^ which revealed the developmental paths from the common neural progenitors to postmitotic SC neurons via the mitotic phase (Figure 3E). To investigate the process of initial dorsal and ventral cell fate commitment, we examined the pseudotemporal transcriptome profiles focusing on the bifurcation point separating the dorsal and ventral branches (Figure 3F). We identified six clusters of differentially expressed genes along the trajectory. The C4 cluster genes (upregulated at the primitive neural progenitors) were functionally associated with glycolysis, whereas the C5 and C6 cluster genes (upregulated along the mitotic dorsal branch) were associated with mitochondrial electron transport, which implied a gradual shift in energy metabolism from glycolysis to oxidative phosphorylation during the transition from neural progenitors to dorsal progenitors.^[^ ^61^, ^62^ ^]^ On the other hand, genes associated with the cell cycle and mitosis were enriched in the C2 cluster (upregulated along the mitotic ventral branch (Data S3, Supporting Information).^[^ ^63^ ^]^ Overall, our single‐cell data with cell type annotation and differentiation trajectory analyses demonstrated that our pSCOs recapitulated the full process of neuronal differentiation in human spinal cords.

### 2.4 Spatial Transcriptomics (ST) of pSCOs

**Figure 4:**
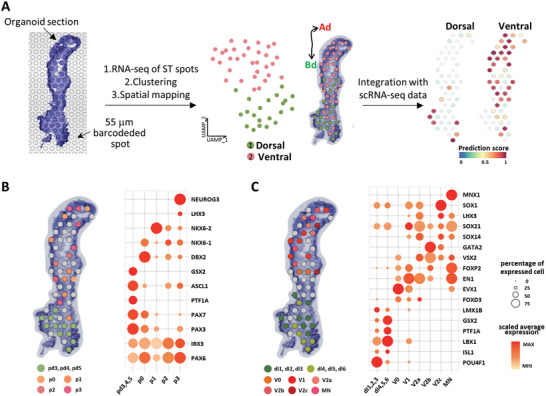
Molecular characterization of the organoid cell population via Spatial transcriptomics (ST). A) Spatial transcriptomic analysis of the DMD10 organoid. Frozen 10 µm sections of DMD10 organoid are placed onto a slide with barcoded spots, and sequencing, clustering, and mapping of 53 spots in the organoid section are done. Two main clusters are mapped with different colors. The dorsal and ventral diagrams on the right side shows the spot assignment by prediction score analysis using integrated scRNA‐seq and ST datasets. Black double arrowhead labeled as Ad and Bd indicates the long axis of the organoid section. Mapping of B) spinal cord mitotic domains and C) postmitotic domains in the organoid section. Spots are differently colored according to their domain assignment, and unspecified spots are colored in gray. Dot plot showing the expression of representative signature genes across assigned domains.

The polarized structure observed in early 3D structure development led us to hypothesize that ventral and dorsal cells identified by the scRNA‐seq analysis were segregated spatially in the organoids. We performed ST analysis to explore the spatial characterization of the organoids using the DMD10 sample (**Figure** **4**A). Two clusters were identified from 53 ST spots where the bottom half (Bd) section expressed genes related to SC dorsal domains, while the top half (Ad) section expressed genes of SC ventral domains. Next, the ST data was integrated with the scRNA‐seq data using an anchor‐based integration method that enables the probabilistic transfer of annotations from the scRNA‐seq data to the ST data.^[^ ^64^ ^]^ We found that dorsal and ventral SC cells identified by the scRNA‐seq analysis were located mainly at the Bd and Ad sides of the organoid section, respectively (Figure 4A). Then, we assigned the detailed cell types of dorsoventral differentiation for each spot. Spots on the Bd side showed marker gene expression of dorsal cell types including pd3‐5 and dI1‐6, whereas marker genes of ventral cell types such as p0‐3 and V0‐2 were largely expressed on the Ad side (Figure 4B,C). Mitotic and postmitotic cell types were observed on both sides. Of note, Mnx1, a marker gene of the MN domain was highly expressed in the Ad spots. Collectively, our ST analysis revealed that our pSCOs have dorsoventral patterning, but rather partially. Nevertheless, our organoids can be broadly specified to be spinal cords since HOX genes were strongly expressed in the entire organoid section (Figure S6, Supporting Information).^[^ ^65^, ^66^ ^]^ Of note, the Bd part exhibited relatively stronger expression of HOX9 genes, which is consistent with our finding that stronger HOX expression was a signature of dorsally confined neural progenitors. Overall, it appears that dorsal and ventral cells in pSCOs were not organized in an anterior‐posterior orientation.

### 2.5 Mapping of Dorsal and Ventral Spinal Cord Domains in the Organoids using Immunostaining

**Figure 5:**
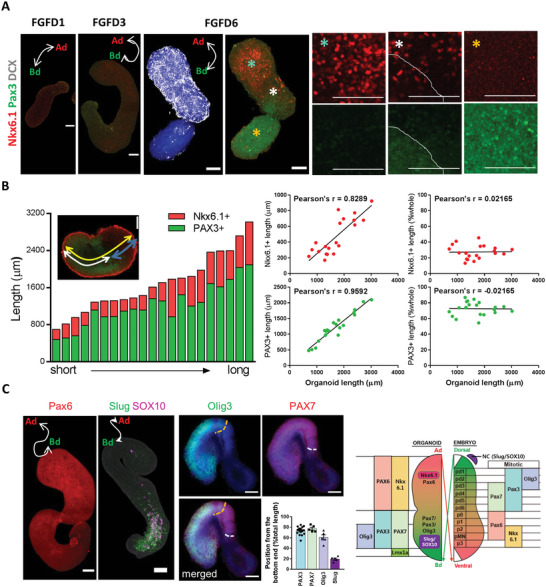
Characterization of spatial dorsoventral patterning of the spinal cord organoid at the mitotic stage. A) Immunofluorescence analysis of spinal cord organoids with dorsal and ventral markers, Pax3 and Nkx6.1, for spinal cord progenitor cells during bFGF treatment. Ad area was specified with DCX‐positive cells. High‐magnification images correspond to colored stars. B) Quantification of the spatial patterning of organoids at FGFD6 based on their axial position. The distance of PAX3+ or Nkx6.1+ area from each pole was measured and plotted in order from shortest to longest (*n* = 21). Pearson's coefficient (*r*) was calculated to determine the correlation between the length of the organoid and the length of each domain (on the left side) and between the length of the organoid and the relative length of each domain (relative to the whole length, expressed as a percentage, on the right side). C) Immunofluorescence analysis of spinal cord organoids with spinal cord progenitor cell and NCC markers during bFGF treatment. Organoids were stained with anti‐Pax6 antibody (DMD2) or costained with anti‐Slug and anti‐SOX10 antibodies (FGFD6). Organoids were costained with anti‐Olig3 and anti‐PAX7 antibodies (DMD2) and the distance of indicated areas from the dorsal pole was measured. Yellow and white dotted lines indicate the border of the Olig3+ and PAX7+ areas, respectively. Quantification at DMD2 shown as mean ± SEM. Diagram on the right side shows that genes expressed in Ad and Bd regions in organoids match mitotic ventral and dorsal markers, respectively, in the embryonic spinal cord during development. White double arrowhead labeled as Ad and Bd indicates the long axis of organoids. Confocal images were taken in 1‐ or 5‐µm steps along the *z*‐axis after fixation and immunostaining with the indicated antibodies and stacked using Z‐stack maximum projection. All images are representative examples from at least three independent experiments. Scale bar, 100 µm.

Consistent with the ST data, we found that Ad parts of pSCOs were gradually developed into Nkx6.1‐positive ventral‐like domains and Bd parts into PAX3‐positive dorsal‐like domains (**Figure** **5**A). Nkx6.1‐ or PAX3‐positive cells at FGFD6 were denser in each pole of the domain and became sparse closer to the border between the two domains. In addition, the earlier appearance of DCX‐positive cells was another hallmark of the Ad part at this earlier time point (Figure 5A). It seemed that the Bd part might need reorganization and stabilization after detachment from a 2D micropattern, which led to later DCX expression in this part compared with the Ad part. Considering the variability of organoid length, we examined whether organoid length affected their cellular composition. We analyzed the spatial distribution of PAX3+ and Nkx6.1+ domains along the axial position of organoids with varying lengths (ranging from 698 to 3018 µm) (Figure 5B and Figure S7, Supporting Information). For this, we measured the distance of PAX3+ or Nkx6.1+ area from each pole and plotted it in order from shortest to longest. We observed that as the length of the organoid increased, the PAX3+ and Nkx6.1+ domains also became longer, as indicated by high Pearson's coefficients (*r* = 0.8289 for PAX3+ and *r* = 0.9592 for Nkx6.1+). When we examined the correlation between organoid length and the relative length (% whole length) of PAX3+ or Nkx6.1+ domains, we found a very weak correlation (*r* = 0.02165 for PAX3+, *r* = −0.02165 for Nkx6.1+). This result indicated that the length of organoids did not have a significant impact on the dorsal and ventral cellular composition of the organoids. Additionally, PAX6 was preferentially found in the Ad part (Figure 5C and Movie S5, Supporting Information), while Slug‐ and/or SOX10‐positive neural crest (NC) progenitor cells were located in the Bd (dorsal) part of the organoids (Figure 5C and Movie S5, Supporting Information). A roof plate marker, Lmx1a was localized at the pole of the Bd part (Figure S8A, Supporting Information). A further separation of domains at the Bd part was observed with the use of other dorsal markers including Pax7 and Olig3, which was similar to what is observed in vivo (Figure 5C). PAX7+ domain covered the entire PAX3+ dorsal part and was bordered by Nkx 6.1+ ventral area. On the other hand, the Olig3+ domain was positioned more dorsally and further away from the ventral border. Interestingly, organoids derived from the micropatterned colony treated with Rac inhibitor failed to organize regionally segregated ventral and dorsal domains, and Nkx6.1‐ and PAX3‐positive cells were randomly intermingled (Figure S8B, Supporting Information). These results suggest that geometric confinement enforced the polarized regionalization of DV domains, and cell type specification is otherwise stochastic.

This DV domain specification could be induced by polarized expression of patterning cues in the organoids, as seen in the spatial transcriptome analysis. For instance, BMP4 and BMP7, which are dorsalizing factors, were enriched in the mitotic dorsal spots in the Bd parts, while cells with high expression levels of ventralizing factor SHH, were found in the postmitotic ventral clusters in the Ad part (Figure S9A, Supporting Information). Accordingly, treatment with LDN, a BMP pathway inhibitor, during bFGF treatment led to the significant shrink of the Pax3‐expressing Bd part with the compensatory expansion of the Nkx6.1‐expressing Ad part (Figure S9B,C, Supporting Information). Conversely, the BMP2 addition resulted in the expansion of the Pax3‐positive Bd part and the disappearance of the Nkx6.1‐positive domain (Figure S9B, Supporting Information). Since BMP signaling is also essential for NC development, NC precursor cells completely disappeared via the inhibition of the BMP signaling pathway; however, they significantly increased upon BMP treatment with uniform distribution along the whole organoid (Figure S9D, Supporting Information). Perturbations of the Shh signaling pathway with antagonist GDC 0449 or agonist purmorphamine (PMP), significantly altered the size of the Pax3‐positive and Nkx6.1‐positive domains (Figure S9B, Supporting Information), indicating that the polarized expression of BMP and SHH signals is responsible for the DV axis formation in pSCO. Additionally, these treatments also affected the growth of organoids, suggesting that cautionary data explanation is yet necessary (Figure S9E, Supporting Information).

The prolonged culture of pSCO allowed the differentiation of domain‐specific postmitotic neurons. While the Ad part began neuronal differentiation earlier than the Bd part, as evidenced by the earlier appearance of DCX+ cells, both parts exhibited simultaneous appearances of postmitotic neuronal markers at around DM 10 days. For instance, the Bd part expressed high levels of Brn3a, a marker for dorsal neurons (dI1‐3) (Figure S10A, Supporting Information), while postmitotic ventral neurons expressing Evx1 (VO), Chx10 (V2a), or Chat (MN) were regionally expressed at the Ad ventral domain (Figure S10A and Movie S6, Supporting Information). Bran3a/Islet‐double positive cells were found at the Bd part (Figure S10A and Movie S6, Supporting Information). On the other hand, Lhx1/2, which has been known to be expressed both in the dorsal and ventral domains in the spinal cord, was expressed in the entire organoids (Figure S10A, Supporting Information). Furthermore, we noticed that the ventral portion as shown in Figure S10A, Supporting Information was substantially reduced compared to the FGFD6 organoids as shown in Figure 5. In fact, reduced ventral proportion in the postmitotic stage was consistent with the spatial transcriptome data that were obtained from DMD10 samples as shown in Figure 4. Thus, it appeared that there were regional differences in the duration of growth. The effect of the disturbance of BMP and Shh signaling pathways on DV patterning was also reproduced in the postmitotic neuron stage (Figure S10B,C, Supporting Information). Together, these results suggest that initial center‐edge patterning spontaneously organizes DV patterning in 3D, which are similar but “upside‐down” fashion of neural patterning in comparison to the in vivo embryonic development (Figure S10D, Supporting Information).

### 2.6 Control of Dorsal/Ventral Proportion by the Micropattern Size Changes

**Figure 6:**
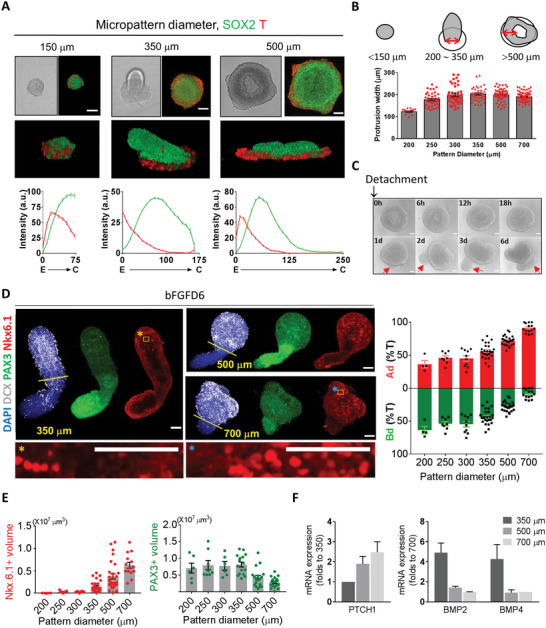
Control of dorsal/ventral proportion in pSCOs by the 2D colony size on micropattern. A) Effects of micropatterned colony size on spatial cell patterning and colony morphogenesis. Immunofluorescence analysis and quantification of colonies grown on micropatterns with the indicated diameters at day 3 with SB/Chir treatment. Quantification of fluorescent intensities at each position shown as mean ± SEM (*n* = total 4795 data points from 15 images at 150 µm, total 15 489 data points from 11 images at 350 µm, total 18 441 data points from six images at 500 µm). B) Quantification of protrusion width at SCD3. The width measured at the top of each colony was indicated by double‐headed red arrows. Protrusion width shown as mean ± SEM. C) Growth of 3D structures derived from a 500‐µm colony after detachment from 2D micropattern in the presence of bFGF for 6 days. Red arrows indicate the extension newly generated from the Bd part. D) Effect of micropatterned colony size on the proportion of Ad/Bd domains of organoids at bFGFD6 and quantification. Ad domain was specified with DCX‐positive cells. High‐magnification images correspond to inset. Quantification of Ad and Bd proportions at each colony size shown as mean ± SEM. E) Quantification of dorsal and ventral domains of organoids depending on the initial 2D micropattern size. Volumes shown as mean ± SEM. F) mRNA levels of PTCH1 and BMPs of pSCOs (FGFD6 and FGFD4, respectively) derived from different sizes of micropatterned colonies. Relative expression level of genes shown as fold changes to 350 or 700 µm colonies (*n* = 3 per group). Confocal images were taken in 1‐ or 5‐µm steps along the *z*‐axis after fixation and immunostaining with the indicated antibodies and stacked using Z‐stack maximum projection. All images are representative examples from at least three independent experiments. Scale bar, 100 µm.

Considering that the center‐edge patterning is the initial step in determining DV patterning, we tested whether the size of the micropattern which affects the center‐edge ratio^[^ ^35^ ^]^ can be used for the control of DV proportion in pSCO. In diameter ranging from 150 to 700 µm, the center‐edge patterning of SOX2/T expression with SB/Chir treatment was observed in all conditions (**Figure** **6**A, and Figure S11A and Movie S7, Supporting Information). However, morphogenic processes were dynamically changed depending on the pattern size. Protrusion observed earlier was only seen in 200–350 µm colonies and donut‐like elevation of cells was seen in 500–700 µm colonies (Figure 6A, and Figures S11B,C and Movie S8, Supporting Information). The width of protrusions in 200–350 µm colonies was, interestingly, similar to that of the donut shapes in 500–700 µm colonies (Figure 6B), suggesting that this morphogenesis may be mediated essentially same cell‐intrinsic properties. After detachment, the Ad part from 500–700 µm micropatterned colonies was also reorganized and expanded, and a small extension started growing from the bottom part (Figure 6C and Movie S9, Supporting Information). Similar to the case of pSCOs, the Ad part could be distinguished by the DCX expression (Figure 6D). When the Ad/Bd volumes were measured in organoids derived from different sizes of micropatterns, the proportion of Ad parts was gradually increased with the increment of initial pattern sizes. Immunostaining with dorsal and ventral markers further confirmed that DV identities of Ad and Bd parts were maintained in organoids derived from larger patterns. Accordingly, as the initial 2D colony size increased, Nkx6.1‐positive ventral domains were increased, and PAX3‐positive dorsal domains decreased in organoids (Figure 6D,E, Supporting Information). Furthermore, the intensity of the Nkx6.1‐postive cells in the ventral part appeared to be stronger in larger‐diameter colonies (Figure 6D). In addition, the changes in DV proportions were maintained at postmitotic stages in longer cultured pSCOs (Figure S12A, Supporting Information). All Bd parts contained Slug‐positive cells (Figure S12B, Supporting Information), which indicated that neural plate border also emerged irrespective of pattern sizes. The expression level of PTCH1, the receptor for SHH, was higher in 500‐ and 700‐µm organoids compared with 350‐µm organoids, and conversely, BMP2 and BMP4 levels were significantly lower in 500‐ and 700‐µm organoids (Figure 6F). Therefore, we speculated that the differences in center‐derived SHH signaling and those in the edge‐derived BMP signaling were involved in the pattern‐size dependent DV pattern control.

### 2.7 Structural and Functional Patterning in Mature pSCOs

**Figure 7:**
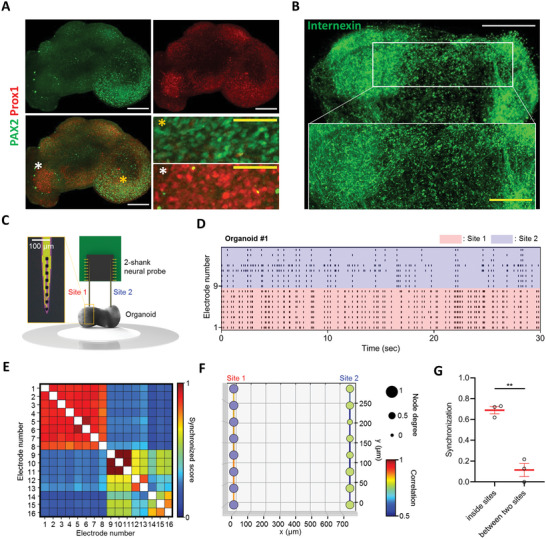
Structural and Functional patterning in mature pSCOs. A) Immunofluorescence analysis of organoids (DMD73) with a dorsal inhibitory neuron marker, PAX2, and late‐born mid and ventral neuron marker, Prox1. Confocal images were taken in 5‐µm steps along the *z*‐axis and stacked using Z‐stack maximum projection (white scale bar, 200 µm, yellow scale bar, 50 µm). B) Immunofluorescence analysis of organoids (DMD73) with anti‐Internexin antibody. Confocal images were taken in 5‐µm steps along the z‐axis and 3D renderings were created (white scale bar, 500 µm, yellow scale bar, 200 µm). All images are representative examples from at least three independent experiments. High‐magnification images correspond to colored stars. C) A schematic diagram illustrating the neural signal recording process from pSCOs using a neural probe with two shanks. The inset image shows the probe's tip with 8 black Pt electrodes. Scale bar, 100 µm. D) Representative raster plot of neural signals recorded from sites 1 and 2 in organoid 1 (DMD73). Specifically, electrodes 1–8 were inserted into site 1 of the pSCO, and electrodes 9–16 were inserted into site 2 of the pSCO. E) The cross‐correlation matrix showing synchronized scores between electrodes in organoid 1. F) The 2D network map displaying connectivity between electrodes in organoid 1. Node color represents the network connection among electrodes. Node degree reflects the number of electrodes connected to each electrode. The line color indicates the correlation between the electrodes. G) Bar plot comparing the degree of synchronization within sites and between two sites (*n* =  3 where *n* is the number of organoids; *t*(4) = 7.961, *p* = 0.0013).  Data are presented as mean values ± SEM with individual data points. Statistical significance was tested with a two‐tailed unpaired *t*‐test. **p < 0.01.

Next, we investigated whether the polarized structure observed in early‐stage spinal cord organoids remained as they matured. Expression of dorsal inhibitory neuron marker, PAX2, and late‐born mid and ventral neuron marker, Prox1, was examined in DMD70 organoids.^[^ ^67^ ^]^ PAX2 expression was localized to the dorsal‐like side of organoids, while Prox1 was more broadly expressed in the organoids with particularly strong expression in the ventral‐like pole (**Figure** **7**A). Furthermore, mature organoids exhibited a dense complex network of neurites on both dorsal‐ and ventral‐like sides, which was clearly marked by the strong labeling with anti‐*α*‐internexin antibody (Figure 7B). It appeared that these networks on both sides were separate from each other with only sparse connections, indicating that distinct neural circuits might be forming within the organoids. To examine whether this structural patterning led to functional patterning, we measured neural signals in both the dorsal and ventral sides of mature organoids (Figure 7C). To achieve simultaneous recordings from both regions, we employed a silicon‐based neural probe with two shanks spaced 750 µm apart (Figure S13A,B, Supporting Information). Utilizing a previously reported protocol,^[^ ^68^ ^]^ we successfully inserted the probe into both regions of the organoid and recorded neural signals in a CO_2_ incubator. Interestingly, we observed asynchronous neural signal patterns between the two regions (Figure 7D, and Figures S13C, and S14A,B, Supporting Information). To examine this more closely, we compared the degree of synchronization between neural signals from both regions and confirmed a low degree of synchronization of 0.217 or less between the two regions (Figure 7E and Figure S14C,D, Supporting Information). Furthermore, to visualize the functional network between two regions, we applied the Louvain algorithm, which is used for graphical network analysis.^[^ ^68^ ^]^ We confirmed the formation of networks within each region but found no significant network formed between the two regions, indicating low functional connectivity between them (Figure 7F and Figure S14E,F, Supporting Information). When we combined the results from measuring three different organoids, we observed a synchronization degree of 0.689 ± 0.03 within each region and a synchronization degree of 0.115 ± 0.06 between the two regions, showing a quantitatively significant difference (Figure 7G). In summary, we demonstrated that functional as well as spatial patternings were developed in our mature spinal cord organoids.

## 3 Discussion

Our study demonstrated that symmetry breaking and spatial patterning by micropatterning in 2D can be used for the polarization of neural organoids in 3D. Although the spatial organization of PSCs resulting from geometry confinement on micropatterns has been reported,^[^ ^32^, ^33^, ^34^, ^35^ ^]^ these studies applied micropatterns to 2D monolayer cultures and did not explore the potential for extending the time over which 3D structures can be cultured. We combined initial 2D micropattern culture with 3D organoid culture and investigated how 2D spatial organization developed into 3D DV‐like features structurally and functionally. Several groups have reported 3D culture systems including gastruloids and organoids that recapitulate symmetry breaking and the characteristic in vivo axis. Autonomous symmetry breaking occurs after bath application of patterning factors to human or mouse PSCs without asymmetry in the initial signal, extra‐embryonic tissues, or localized signaling centers.^[^ ^20^, ^21^, ^22^, ^23^, ^24^ ^]^ On the other hand, to establish an axis in the neural organoids, either the addition of signaling factors to the media or a fusion of signaling‐releasing cells with forebrain organoids is necessary.^[^ ^13^, ^14^, ^15^, ^25^, ^28^ ^]^ Compared with the above cell patterning strategies, a key feature of our strategy was that we utilized a very simple, easy, and reproducible micropattern technique for initial 2D cell patterning. Geometric confinement by micropattern is sufficient to induce spatial organization that developed into well‐ordered spatial positional domains in our pSCO. And the change in micropattern sizes enabled us to control the proportion of different domains in 3D, showing the flexibility of our system. Spatial patterning on micropattern has been explained using the “reaction‐diffusion” or “edge‐sensing” concepts.^[^ ^36^, ^69^ ^]^ Recently, we also demonstrated that the difference in the apical specialization depending on the cell position in the colony altered drug permeability, explaining how edge‐sensing can occur.^[^ ^70^ ^]^ The relocalization of *β*‐catenin in response to SB/Chir was different at the edge and center cells and T‐positive cells were generated at the edge of colonies irrespective of colony size in our study. Therefore, spatial patterning of SOX2‐ and T‐positive cells in our system might be better explained by the “edge‐sensing” model.

The difference between edge and center cells in the colony was also demonstrated in terms of physical force. The force map indicated that the combination of the inward pulling force in edge cells and high tension at the colony center might develop the force for cells to sprout up. Notably, an increase in the micropattern size evoked the switch of morphogenesis processes from single‐center protrusion to doughnut‐like elevation, although the edge‐specific cell specification was observed regardless of the pattern size. Karzbrun et al. also reported that micropattern size can control morphogenesis.^[^ ^41^ ^]^ They found that changes in the width of the neural plate due to different micropattern sizes led to the different morphogenesis of the neural tube. In fact, single protrusion and doughnut‐like elevation showed common features: both appeared to be associated with the pulling force from the edge cells based on the edge‐sensing model, and the width of the protrusion/elevation was constant. These results suggested that the collective behavior of cells for morphogenesis is dependent on the self‐organizing properties of cells. Thus, these results demonstrated that the cell type specification and the morphogenesis are dissociable events. In our pSCO model, axial elongation or doughnut elevation was associated with the DV axis in our pSCO model, while in the gastruloid and neural tube organoid models, axial elongation was more associated with AP axis formation,^[^ ^21^, ^24^, ^59^, ^71^ ^]^ which seems to be more relevant to in vivo development. Considering that the in vitro organoid model lacks non‐neural components which might be essential for the in vivo neural morphogenesis, our data suggest that the neural intrinsic, self‐organizing properties are insufficient for the in vivo‐like morphogenesis. Collectively, these results suggest the interplay of cell type specification with self‐organizing morphogenetic responses to the environments are dissociable but precisely coordinated during embryonic development in vivo. Although the protrusion/elevation morphogenesis observed in our system is yet difficult to be linked with in vivo morphogenesis, it may be associated with different timing of NMP‐derived neural progenitors along the dorsoventral axis,^[^ ^72^ ^]^ and “oblique” formation of junctional neurulation in the caudal spinal cord.^[^ ^73^ ^]^

Detachment of initially patterned 2D cell colonies in micropatterns eventually resulted in the 3D pSCO development. BMP and Shh are DV patterning factors in vivo, and several lines of evidence demonstrated that the same factors are involved in the DV axis formation in pSCO. For instance, BMP and Shh were expressed in dorsal and ventral clusters, respectively, as shown in the scRNA‐seq analysis and spatial transcriptomics (Figure S9A, Supporting Information). Two regions in the organoid were also distinguished by the different rosette structures and emergence times of DCX‐positive cells (Figures 2B and 5A, Supporting Information). Rosette structures have been reported to differ according to the distance from the Shh source in forebrain organoids,^[^ ^28^ ^]^ which is consistent with our current observations. As shown in Figure S10D, Supporting Information, our organoids appeared to partially recapitulate the mediolateral patterning of the neural plate in vivo in many aspects. Neuroectoderm or neuroepithelial cells that cover the surface of 3D structures and form neural rosettes inside organoids sprout up and become ventral parts like ventral part formation from the neural plate at the neural tube fold stage (second row in Figure S10D, Supporting Information). After neural tube closure, the dorsal neural tube is isolated from the non‐neural ectoderm in vivo. Similarly, the base part of the 3D structures detached from the culture plate becomes dorsal domains in organoids (third row in Figure S10D, Supporting Information). As NC cells delaminate from the region between the dorsal neural tube and overlying ectoderm in vivo, they are generated in the dorsal‐most part of the organoid (four rows in Figure S10D, Supporting Information). Finally, neural stem cells at the top part of the organoids become ventral progenitor cells, and cells at the bottom become dorsal cells, leading to dorsal and ventral postmitotic neurons with spatial DV organization reminiscent of the spinal cord DV axis in vivo (bottom row in Figure S10D, Supporting Information).

After extended culture, pSCOs matured and established anatomically and functionally specialized local circuits. The dorsal horn of the spinal cord mediates sensory processing and consists of projection neurons and a complex network of excitatory and inhibitory interneurons, while the ventral region forms different connectomes to regulate motor functions and contains a diverse network of interneurons and motor neurons.^[^ ^74^, ^75^, ^76^ ^]^ Particularly, ventral interneurons that form complex interconnections with each other, assemble into a neuronal network called the central pattern generator (CPG) that drives rhythmic motor patterns. This ventral network has been successfully recapitulated by organoid models. The circuitoid model, which includes ventral interneurons and motor neurons derived from mouse ESCs, exhibited rhythmic bursts of activity that closely resembled the in vivo CPG.^[^ ^77^ ^]^ The trunk neuromuscular organoid model has been also reported to develop CPG‐like microcircuits.^[^ ^2^ ^]^ Whereas these two models generated the ventral CPG, our pSCOs developed dorsal and ventral neuronal networks innately in their respective regions. Neural circuits in the mature human spinal cords originate from the polarized alignment of neural progenitors in early development, while these progenitor cells undergo significant amounts of cell migration during maturation. Although the pSCO showed less obvious unipolar dorsal and ventral regionalization in their maturity, they maintained anatomical and functional arealization. These features were in sharp contrast to the unpolarized SCOs without any axis, which displayed a highly connected axonal trajectory map and synchronized neural activity.^[^ ^6^ ^]^ Therefore, we speculate that the polarization of the organoids with early patterning can influence the later sophisticated organization of neural circuits. Accordingly, this strategy may be more suitable for modeling developmental disorders related to proper neural circuit formation and investigating the basic biology of human neural development. Recently, many efforts to profile the cell types of the adult spinal cord have been made, taking advantage of scRNA sequencing or spatial transcriptome techniques. It has been shown that dorsal and ventral neurons in the adult display no longer region‐specific markers of the early developmental stage and that they require differential levels of gene expression related to their functions.^[^ ^67^ ^]^ Therefore, to understand how spinal neuron populations in the dorsal and ventral regions are organized to proper functions during maturation, it is crucial to establish a link between their transcriptional identity in the mature stage with neuronal connectivity leading to functional neuronal networks. We believe that our pSCOs could be a valuable tool for investigating how region‐specific interneuron networks establish and integrate as spinal cord neurons mature, combining their transcriptome profiling, electrophysiological properties, and morphologic and neurochemical features.

Our strategy to make DV patterning in organoids, however, had a few limitations and remaining issues that need to be addressed in future studies. For instance, while we found a rich repertoire of spinal cord cells existed and was spatially organized, signaling gradient‐dependent organization of dorsoventral domains, as shown in embryonic spinal cord development, was rather limited. Furthermore, neural folding morphogenesis was not clearly seen in this model, which is in contrast to the 3D reaggregation‐based unpolarized SCOs.^[^ ^6^ ^]^ Thus, further refinement and the ways to induce proper local arealization signals may be the next step to be addressed.

## 4 Experimental Section

### Cell Culture and Differentiation

The study protocol was approved by Korea University Institutional Review Board (KUIRB), Korea University. All experiments were performed under the guidelines and regulations of KUIRB. For maintenance, hESCs (H9) were grown in the E8 or mTeSR1 (STEMCELL Technologies) medium in 35 mm‐diameter tissue culture dishes coated with Matrigel (Corning, 354277; 1:25 in DMEM/F12). Cells were passaged using ReLeSR (STEMCELL Technologies) every 5 days. For experiments, hESCs were detached from 35 mm‐diameter dishes, and the cells were softly resuspended in 2 ml of mTeSR1 to keep them as aggregates, with a total cell number of generally around 5 × 10.^6^ One hundred microliter of cell aggregate mixture was added to each well of a 12‐well micropattern plate. The following day, the medium was changed with fresh mTeSR1 medium in the morning, and micropatterns were generally filled with cells at least 50% in the afternoon. mTeSR medium was replaced with the neural cell induction medium (DMEM/F12, 1% N2 supplement, 2% B27 supplement, 1% NEAA, 1% penicillin, and 0.1% 2‐mercaptoethanol) containing 10 µM SB431542 (R&D) and 3 µM CHIR99021 (Sigma), and was subsequently replaced every day. After 3 days, the medium was removed, and a cell recovery solution (Corning, Cat#.354253) was applied to the coverslips. The coverslips were then incubated on ice for 10 min. Further, colonies were detached using the pressure of the solution from the pipette under a stereoscopic microscope and transferred into an ultra‐low attachment 96‐well clear round bottom plate (Corning‐Costar). They were further grown in 3D with the neural cell induction medium containing 20 ng mL^−1^ bFGF. After 6 days of bFGF treatment, 3D structures were grown without bFGF in the neural cell induction medium, which we called the differentiation medium (DM) stage. An all informative list of reagents including antibodies is shown in Table S1, Supporting Information.

### Stamp Fabrication and Micro‐Contact Printing

A stamp for printing biomolecules was fabricated using soft lithography techniques.^[^ ^78^, ^79^ ^]^ The photoresist (SU‐8 2010, Microchem, USA) was spin‐coated for 30 s at 1000 rpm (target thickness: 20 µm) and baked for 4 min at 95 °C. The photoresist layer was exposed to UV light using a pre‐designed photomask (exposure dose: 175 mJ cm^−2^). After exposure, the wafer was baked for 5 min at 95 °C. The wafer was then immersed in a SU‐8 developer to remove the uncrosslinked photoresist layer. After the developmental process, the wafer was sequentially rinsed with isopropanol and distilled water. To cast the stamp, the polydimethylsiloxane (PDMS) prepolymer:curing agent mixture (10:1 [w/w], Sylgard 184 Silicone Elastomer Kit, Dow Corning, USA) was cured overnight on the SU‐8 mold at 60 °C. Finally, the cured PDMS was cut into an appropriate size to obtain the finished PDMS stamps. Microstamps with the desired dot pattern were inked with Matrigel (Corning, 354277; 1:10 in DMEM/F12) and then applied to the coverslips for printing.^[^ ^80^ ^]^

### Immunostainings

Cells grown on coverslips were fixed using 4% paraformaldehyde in 0.1 M phosphate‐buffered saline (PBS; pH 7.4) for 20 min at 25 °C. Cells were washed three times with PBS and then blocked with 0.2% Triton X‐100 with 3% bovine serum albumin (BSA) in PBS for 30 min at 25 °C. Subsequently, cells were incubated with primary antibodies in a blocking buffer overnight at 4 °C. After incubation, the cells were washed three times with PBST (0.2% Triton X‐100 in PBS) and afterward incubated with secondary antibodies, with Hoechst for cell nuclei staining (1:2000), in the blocking buffer for 1 h at 25 °C. After washing with PBST, the cells were imaged using a confocal microscope (Leica TCS SP8 confocal microscope).

3D structures were fixed using 4% paraformaldehyde in 0.1 M PBS (pH 7.4) for 1 h at 25 °C, washed three times with PBS, blocked with 0.1% Triton X‐100 with 6% BSA in PBS overnight at 25 °C, and then incubated with primary antibodies in the blocking buffer overnight (twice) at 25 °C. After incubation, the cells were washed three times with PBST (0.1% Triton X‐100 in PBS) and subsequently incubated with secondary antibodies with Hoechst (1:2000) in the blocking buffer overnight (twice) at 25 °C. After washing with PBST, organoids were mounted onto the dishes (µ‐dish, Ibidi) with a mounting solution (25% urea and 65% sucrose in H_2_O) for optical clearing and imaged using a confocal microscope (Leica TCS SP8 confocal microscope).

### Time‐Lapse Imaging

Morphogenesis of 3D structures was recorded using the JuLI stage (NanoEnTek Corp.), a real‐time cell history recorder. Cell images were acquired directly from the culture plate (12 wells) in an incubator automatically at a bright field. A 4× objective lens was used, and the focus was sustained.

### Quantitative Analysis of Kinematics and Mechanical Dynamics of hESC Colony

The movement and physical force of the cell colonies were measured as previously reported.^[^ ^81^ ^]^ Briefly, hESCs were patterned on polyacrylamide (Bio‐Rad, USA) substrates embedded with fluorescent beads (diameter = 500 nm; FluoSpheres; Invitrogen, USA) in the same manner as described above. Using a JuLI stage live cell imaging system (NanoEnTek, Korea) with a 4× objective lens (Olympus, Japan) in a cell culture incubator, a bright‐field channel for the patterned cell colony images and a red fluorescent protein (RFP) channel for bead images were simultaneously collected while culturing the hESC colony.

The obtained images were numerically converted and calculated using a customized source code developed with MATLAB (MathWorks Inc., USA). To calculate the cell and bead displacements, particle image velocimetry analysis was conducted on each image set. The displacement result from the bright field images was converted to a movement trajectory and velocity of the cells within the colony, and the displacement result from the RFP images was converted into the traction force of the hESCs using unconstrained Fourier transform traction microscopy. The traction data were used to calculate the tensional stress within the colony using monolayer stress microscopy.

### Microarray Analysis

Total RNA was extracted using TRIzol from the pooled samples of six stages including ES, SCD2, SCD3, FGFD3, FGFD6, and DMD8. RNA purity and integrity were evaluated using the OD 260/280 ratio and analyzed using the Agilent 2100 Bioanalyzer (Agilent Technologies, Palo Alto, USA). According to the manufacturer's protocol, the Affymetrix Whole transcript Expression array process was performed (GeneChip Whole Transcript PLUS reagent Kit). First, cDNA was synthesized using the GeneChip whole transcript (WT) amplification kit as described by the manufacturer. Afterward, using the GeneChip WT Terminal labeling kit, the sense cDNA was fragmented and biotin‐labeled with terminal deoxynucleotidyl transferase. At 45 °C, ≈5.5 µg of labeled DNA target was hybridized to the Affymetrix GeneChip Human 2.0 ST Array for 16 h. After washing, hybridized arrays were stained on a GeneChip Fluidics Station 450 and scanned using a GCS3000 Scanner (Affymetrix). Signal values were computed using the Affymetrix GeneChip Command Console software. The data were subsequently normalized using the robust multi‐average (RMA) method implemented in Affymetrix Power Tools. The result was exported with gene‐level RMA analysis, and the differentially expressed gene analysis was employed. The statistical significance of the expression data was determined by fold change. The authors focused on genes variable along cellular differentiation toward spinal cord development. Variable genes were identified by calculating the median absolute deviation (MAD) across all samples with the cutoff of the top 5% of the MAD values, which resulted in ≈1400 genes. These genes were then divided into two clusters, up‐and down‐regulated genes on differentiation, which were used for the analysis of functional enrichment in the GO terms for biological processes. For enrichment analysis, Enrichr's web‐based enrichment analysis tool^[^ ^51^, ^52^ ^]^ was used to analyze the 250 most variable genes across samples.

### scRNA‐seq Analysis

Samples of organoids at the FGFD1, FGFD6, and DMD10 stages were pooled and chopped into small pieces. Organoid pieces were then digested in dispase for 10 min at 37 °C and dissociated into single cells. Further, the chromium controller was used to prepare libraries according to the 10× Single Cell 3’ v3 protocol (10x Genomics, Pleasanton, USA). After washing, cells were diluted in nuclease‐free water to achieve a targeted cell count of 10 000 and mixed with a master mix. The mixture was loaded with Single Cell 3′ gel beads and partitioning oil into a Single Cell 3′ Chip. RNA transcripts from single cells were uniquely barcoded and reverse‐transcribed within the droplets. Subsequently, cDNA molecules were pooled, and the cDNA pool went through an end repair process, the addition of a single “A” base, and ligation of the adapters. The products were then purified and enriched with PCR to create the final cDNA library. The purified libraries were quantified using qPCR according to the qPCR quantification protocol guide (KAPA) and qualified using the Agilent Technologies 4200 TapeStation (Agilent Technologies). Subsequently, the libraries were sequenced using HiSeqX (Illumina) with a read length of 28 bp for read 1 (cell barcode and UMI), 8 bp index read (sample barcode), and 91 bp for read 2 (RNA read).

Single‐cell gene expression data was analyzed using Cell Ranger v5.1.0. Briefly, raw BCL files from Illumina sequencing instruments were demultiplexed to generate FASTQ files using “cellranger mkfastq.” These raw FASTQ files were then analyzed using “cellranger count.” The “cellranger count” step included mapping to the human reference genome (GRCh38), measuring gene expression with the unique molecular identifier (UMI) and cell barcode, determining cell clusters, and conducting differential gene expression analysis. The final dataset was built by aggregating multiple independent samples using “cellranger aggr.”

Raw count matrices of single‐cell data were imported into Seurat 4.0.2.^[^ ^82^ ^]^ Further analysis was performed using R functions unless otherwise noted. For quality control, the authors filtered out from downstream analysis i) genes expressed in less than 10 cells, ii) cells with over 15% reads mapping to mitochondrial genes to avoid dying cells, and iii) cells with more than 8000 or fewer than 200 uniquely detected genes in each cell to discard data from empty droplets or doublets. 10 618, 11 690, and 13 696 cells remained from the FGFD1, FGFD6, and DMD10 samples respectively, and were used for further analysis. The raw count values of each sample were then log‐normalized using logNormCounts::scater^[^ ^83^ ^]^ and then scaled using ScaleData::Seurat with default parameters.

Next, RunHarmony::harmony was used to remove variations from different runs for each sample without losing biological variations. The highly variable genes (HVGs) were selected using getTopHVGs::scran^[^ ^84^ ^]^ for cell clustering. The genes with false discovery rates (FDR) < 0.01 were identified as HVGs. Cell clustering was performed with FindCluster::Seurat using the top 20 principal components (PCs) of 1424 HVGs, and the result was visualized using uniform manifold approximation and projection (UMAP) version 0.5.1.

### Cell‐Type Annotation

Cell‐type annotation was carried out for the 20 clusters previously obtained. First, the authors tried to annotate the cluster with the expression of conventional markers following the snapshot diagram of spinal cord development by Alynick et al.^[^ ^55^ ^]^ Next, for an unbiased cell type assignment, Seurat v3^[^ ^64^ ^]^ data integration and label transfer methods were used, following the same protocol used in the previous study.^[^ ^6^ ^]^ Briefly, the cell‐type assignment was performed in a two‐step process using the “Mouse Spinal Cord Atlas” dataset as the reference. The coarse‐level assignment identified progenitor cells and neurons, whereas the fine‐level analysis included all dorsal/ventral subclasses (Figure S5, Supporting Information). Cell types with the highest prediction score were assigned to each cluster initially. The expression of known marker genes of the assigned cell type for each cluster was then examined. For clusters with concordant gene expression patterns, the subclass assignment was maintained. Otherwise, the authors annotated it out at the coarse level. Default parameters and 30 PCs were used in all Seurat functions.

### Trajectory Analysis

To elucidate the differentiation processes of cells in the organoid, a single‐cell trajectory analysis was performed using Monocle3 (version 1.0.1) that inferred an evolutionary trajectory and placed cells at proper positions along the trajectory (pseudotime).^[^ ^60^ ^]^ The normalized data from Seurat was used as input to Monocle3. First, the cells were clustered using the cluster_cells() function to separate partitions assuming the common transcriptional ancestry, which predicted only one partition in the dataset. Next, the learn_graph() function was used to fit the principal graph and then ordered the cells using the order_cells() function. A point with the most FGFD1 samples was chosen as the starting point.

To identify genes that determined the dorsal and ventral fates during early differentiation, the subset of the branches belonging to the neural progenitor cluster was taken. The differentiation of neural progenitors into mitotic dorsal and mitotic ventral axes along the trajectory in an outgoing way (from the middle to both sides) was observed. Then, the authors obtained genes that exhibited differential expression in the pseudotime along the trajectory using the graph_test() function. FDR < 0.01 yielded 1617 statistically significant genes, which were further grouped into six clusters according to the pseudotemporal expression pattern.

### Spatial Transcriptome Analysis

Frozen samples were embedded in an OCT compound (VWR, USA) and sectioned at −20 °C with a cryotome (Thermo Scientific, USA). Tissue sections were placed on chilled Visium Tissue Optimization (1000193, 10X Genomics, USA) and Visium Spatial Gene Expression (1000184, 10X Genomics), and attached to the slides by warming on a heating block. The sections were fixed onto the slides in chilled methanol for staining under the Visium user guide (10X Genomics, USA). The slide was covered with library spots; each spot (55 µm) in this study contains ≈15 cells. For gene expression analysis, cDNA libraries were prepared according to the Visium Spatial Gene Expression User Guide and sequenced on a NextSeq 550 System (Illumina, USA) at a sequencing depth of up to 250 M read‐pairs per sample.

Raw FASTQ files and histology images were processed using the Space Ranger software v1.0.0, which uses STAR v.2.5.1b^[^ ^85^ ^]^ for genome alignment against the Cell Ranger hg38 reference genome “refdata‐cellranger‐GRCh38‐3.0.0,” available at: http://cf.10xgenomics.com/supp/cell‐exp/refdata‐cellranger‐GRCh38‐3.0.0.tar.gz. The alignment and count processes were performed by “spaceranger count,” which commenced with specifying the input of FASTQ files, reference, section image, and Visium slide information. The pipeline detects a tissue area by aligning the image to the printed fiducial spot pattern of a Visium slide and recognizing stained spots from the image.

The Visium spatial transcriptome data were analyzed by closely following the computational pipeline for standard single‐cell transcriptome data. The raw count matrix of VISIUM data was loaded into Seurat 3.2.1. The authors then discarded spots of low quality that contained more than 6% of their reads mapping to mitochondrial genes (dying cells) or that had less than 2000 uniquely expressed genes (insufficient cells). The remaining 53 spots were assumed to be of good quality and used in the downstream analysis. After normalization and scaling, spots were clustered using the 20 most significant PCs as before, which yielded two clusters of spots. In an effort to characterize these two clusters, a composite analysis was performed that integrated the spatial transcriptomic data with scRNA‐seq data as follows.

1. scRNA‐seq data for the DMD10 sample that matched the spatial transcriptome data were used as a reference. Thus, the scRNA‐seq data were reprocessed for the DMD10 sample separately and obtained cell clusters. Annotations of cell states (mitotic or postmitotic), dorsal and ventral axis, and sections in each state were carried out in the same way as before.
2. The label transfer process was performed by following the “reference‐based” integration workflow of Seurat. Briefly, each dataset was normalized with SCTransform and integrated into two datasets using FindTransferAnchors. Subsequently, the discrete labels from scRNA‐seq clustering and annotation were transferred to the spatial transcriptomic data using the TransferData function of Seurat.
3. The above procedure assigned a probabilistic score (between 0 and 1) of each scRNA‐seq‐derived label for each spot. Thus, a series of probabilistic scores representing diverse cell states and sections were obtained, which could be used to characterize each spot in the spatial transcriptome data.

Each spot was further annotated according to the marker genes in the Snapshot on the spinal cord development.^[^ ^55^ ^]^ Each spot was assigned to a specific section if the scaled expression of a unique section marker was greater than 1.

### Electrophysiology

A 2‐shank silicon‐based neural probe with 16 black Pt electrodes was utilized to record neural signals from mature organoids. The fabrication and packaging of the neural probe, along with black Pt plating, were carried out using previously established methods.^[^ ^68^ ^]^ To simultaneously measure neural signals from the dorsal and ventral sites of the mature organoid, a multi‐shank neural probe with a shank distance of 750 µm was used. Each shank had a size of 56  × 20 µm to minimize tissue damage during insertion into the organoid. The electrode size was 14 × 14 µm^2^, and the distance between electrodes was 40 µm, enabling the recording of signals from neurons ≈300 µm deep.

To record neural signals from the organoids, the packaged probe with two 1 mm screws on a mechanical structure was initially secured for positioning adjustment in the depth direction.^[^ ^68^ ^]^ A 35 mm petri dish was fixed to the bottom of the structure using double‐sided tape and placed the organoid in the center of the dish. The probe was slowly inserted into the organoid using the mechanical structure. To avoid rapid evaporation of the culture medium, the entire structure was placed in a small acrylic box and filled in the petri dish with 1.5 mL of fresh DMEM/F12‐based culture medium. The entire structure was subsequently placed in a CO2 incubator set at 37 °C and 5% CO2. Neural signals were recorded by electrically connecting the probe to an external commercialized measuring equipment (RHD2132 amplifier board connected to an RHD2000 Evaluation System, Intan technologies, USA) and utilizing the provided software with the following settings: 20 kS·s^−1^ per channel, 300 Hz high pass filter, 6 kHz low pass filter, 16‐bit ADC.

After recording spontaneous neural signals from three organoids, a custom spike sorting algorithm was utilized previously reported to detect the neural signals.^[^ ^86^ ^]^ The threshold amplitude to 60 µV was set, which is three times higher than the noise level of 20 µV. The detected signals were then displayed as a raster plot. The degree of synchronization between electrodes was also analyzed using Pyspike (https://github.com/mariomulansky/PySpike), and the results were presented as a color‐mapped cross‐correlation map. Finally, Finally, based on the analyzed synchronization degree, a graphical network analysis was carried out using previously reported custom code^[^ ^68^ ^]^ and visually represented the network results as a 2D map.

### Image Analysis and Statistical Analysis

Every experiment was repeated at least three times and representative images were shown in Figures. Image analysis was performed with Custom MATLAB (R2017a, MathWorks) and ImageJ. Chart drawing and statistical analyses were performed using the SigmaPlot software and Graphpad Prism. All data were presented as mean ± SEM. For two group comparisons, a two‐tailed Student's *t*‐test was used. For comparisons of more than two groups, One‐way analysis of variance (ANOVA) with Tukey's post hoc test was used. A *p* < 0.05 was considered to be statistically significant.

## Conflict of Interest

The authors declare no conflict of interest.

## Author Contributions

K.S. and S.C. contributed equally to the study. K.S. and J.R.R.: Conception, design of the work, acquisition, analysis, interpretation of data, drafting, and revision. S.C. and S.L.: Analysis, interpretation of data, and drafting. H.S. and I.C.: Acquisition, analysis, interpretation of data, and drafting. A.S. and B.L.: Acquisition. J.‐H.L., Y.K., H.M.C., Y.J.K., E.Y., D.G., and H.K.: Resources. J.H.K.: Analysis. H.J.: Acquisition, analysis, and interpretation of data. Y.P.: Interpretation of data. H.Y.K., T.L., and W.‐Y.P.: Acquisition. W.S.: Conception, design of the work, interpretation of data, draft, and revision.

## Supporting information

Supporting Information

Supplemental Movie 1

Supplemental Movie 2

Supplemental Movie 3

Supplemental Movie 4

Supplemental Movie 5

Supplemental Movie 6

Supplemental Movie 7

Supplemental Movie 8

Supplemental Movie 9

## Data Availability

The data that support the findings of this study are available from the corresponding author upon reasonable request.
