# Supplementary material for: Symmetry Breaking of Human Pluripotent Stem Cells (hPSCs) in Micropattern Generates a Polarized Spinal Cord‐Like Organoid (pSCO) with Dorsoventral Organization
Source: Adv Sci (Weinh). 2023 May 12;10(20):2301787. doi: 10.1002/advs.202301787 (PMC10369253; doi:10.1002/advs.202301787)
Supplement: Supplementary file 1 — Supporting Information [file ADVS-10-2301787-s002.pdf]

ADVANCED SCIENCE
Open Access

Supporting Information

for *Adv. Sci.*, DOI 10.1002/advs.202301787

Symmetry Breaking of Human Pluripotent Stem Cells (hPSCs) in Micropattern Generates a Polarized Spinal Cord-Like Organoid (pSCO) with Dorsoventral Organization

Kyubin Seo, Subin Cho, Hyogeun Shin, Aeri Shin, Ju-Hyun Lee, June Hoan Kim, Boram Lee, Hwanseok Jang, Youngju Kim, Hyo Min Cho, Yongdoo Park, Hee Youn Kim, Taeseob Lee, Woong-Yang Park, Yong Jun Kim, Esther Yang, Dongho Geum, Hyun Kim, Il-Joo Cho, Sanghyuk Lee, Jae Ryun Ryu and Woong Sun**

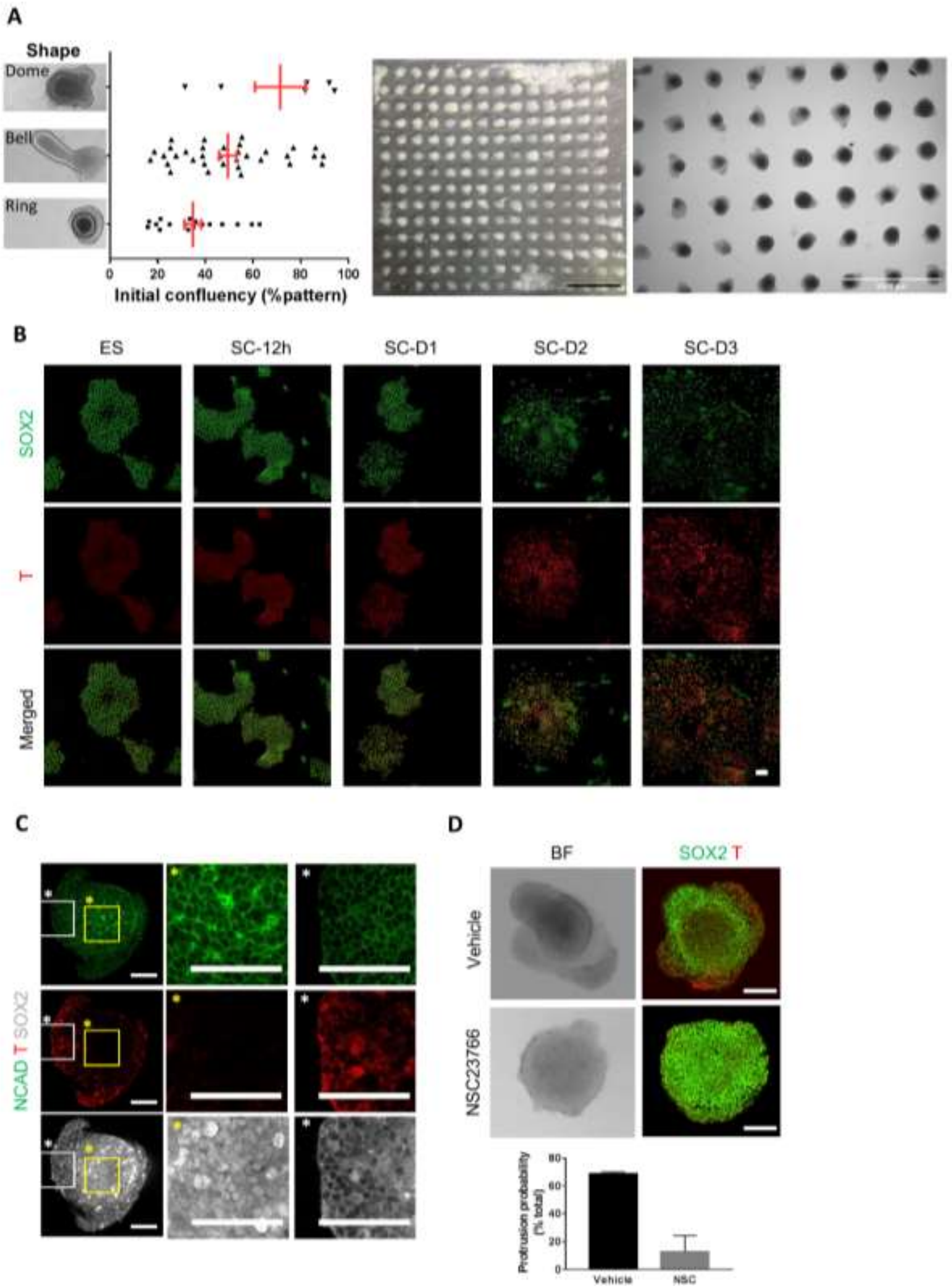

Figure S1. Characterization of differentiated hESCs. (A) Effect of initial confluency of micropatterned hESC colonies (D0) on colony morphogenesis at SC-D3. Quantification of the initial confluency (D0) at each colony (% pattern) shown as mean ± SEM. When colonies

were filled more than 50 % with cells, but not completely, the yield of center protrusion was around 95 %. Representative images of differentiated colonies at SCD3 at low magnification (black scale bar, 2 mm) and high magnification (white scale bar, 2 mm) (B) Time-course immunofluorescence analysis of monolayer hESC differentiation upon SB/Chir treatment under standard culture. Merged images shown at the bottom row. (C) NCAD expression in micropatterned colonies treated with SB/Chir at Day 3. High resolution images correspond to colored stars. (D) Effect of NSC23766, Rac inhibitor, on spatial cell patterning. NSC23766 or vehicle was added to micropatterned colonies at SC-D2. Protrusion probability shown as mean ± SEM (n = 13 per group). Confocal images were taken in 5-μm steps along the z-axis after fixation and immunostaining with the indicated antibodies, and three-dimensional renderings were created. All images are representative examples from at least three independent experiments. Scale bar, 100 μm.

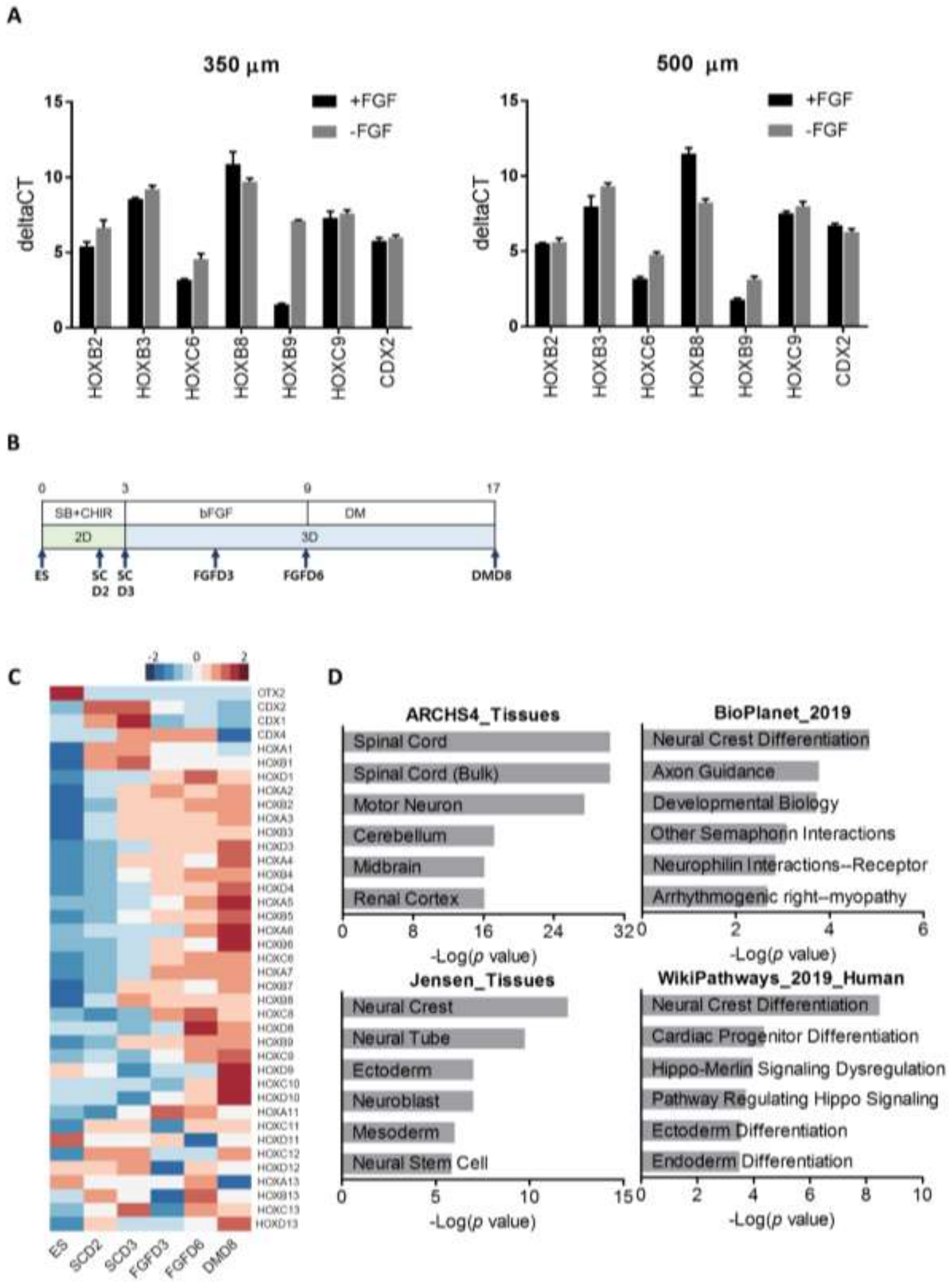

Figure S2. Microarray analysis of temporal changes. (A) Effect of FGF on posteriorization of 3D structures. 3D structures were cultured in the presence or absence of FGF and pooled samples at FGFD6 were used for real time PCR assay (n = 3 per group). (B) Schematic of the

time points at which samples for microarray analysis were collected. Pooled samples at each group were used for microarray analysis. (C) Heatmap of HOX gene expression in organoids over time. (D) Enrichr analysis of Top 250 most variable genes.

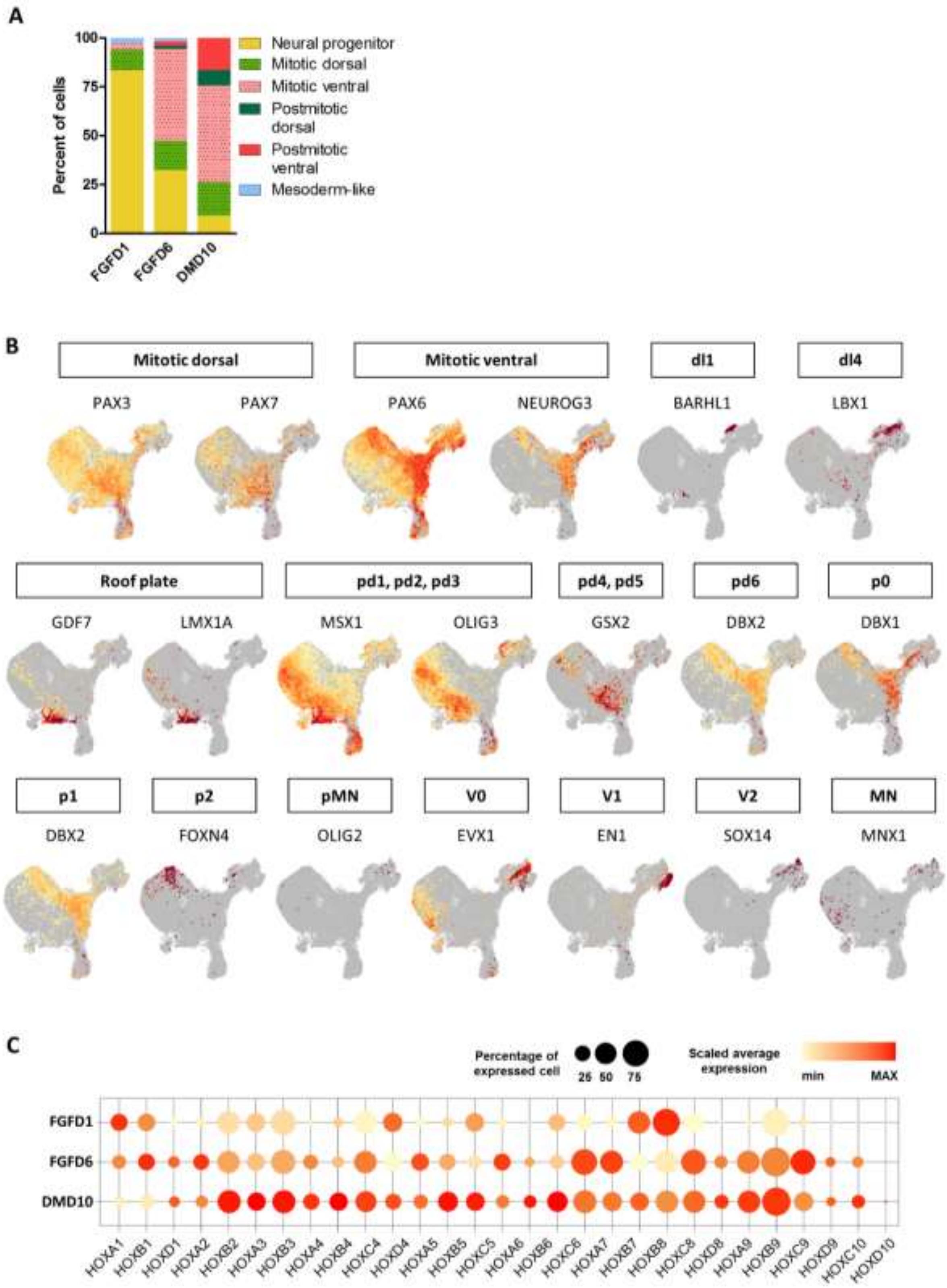

Figure S3. Single cell RNA-sequencing analysis. (A) Percentage of cells of each cluster in FGFD1, FGFD6 and DMD10 cells are differently colored. (B) Feature plots of representative marker genes used to assign cluster identities colored based on gene expression level. (C) Dot

plots of posterior specific HOX genes across samples. The size of each circle indicates the percentage of cells where the indicated gene was detected, and the color intensity reflects the scaled average expression level within cells.

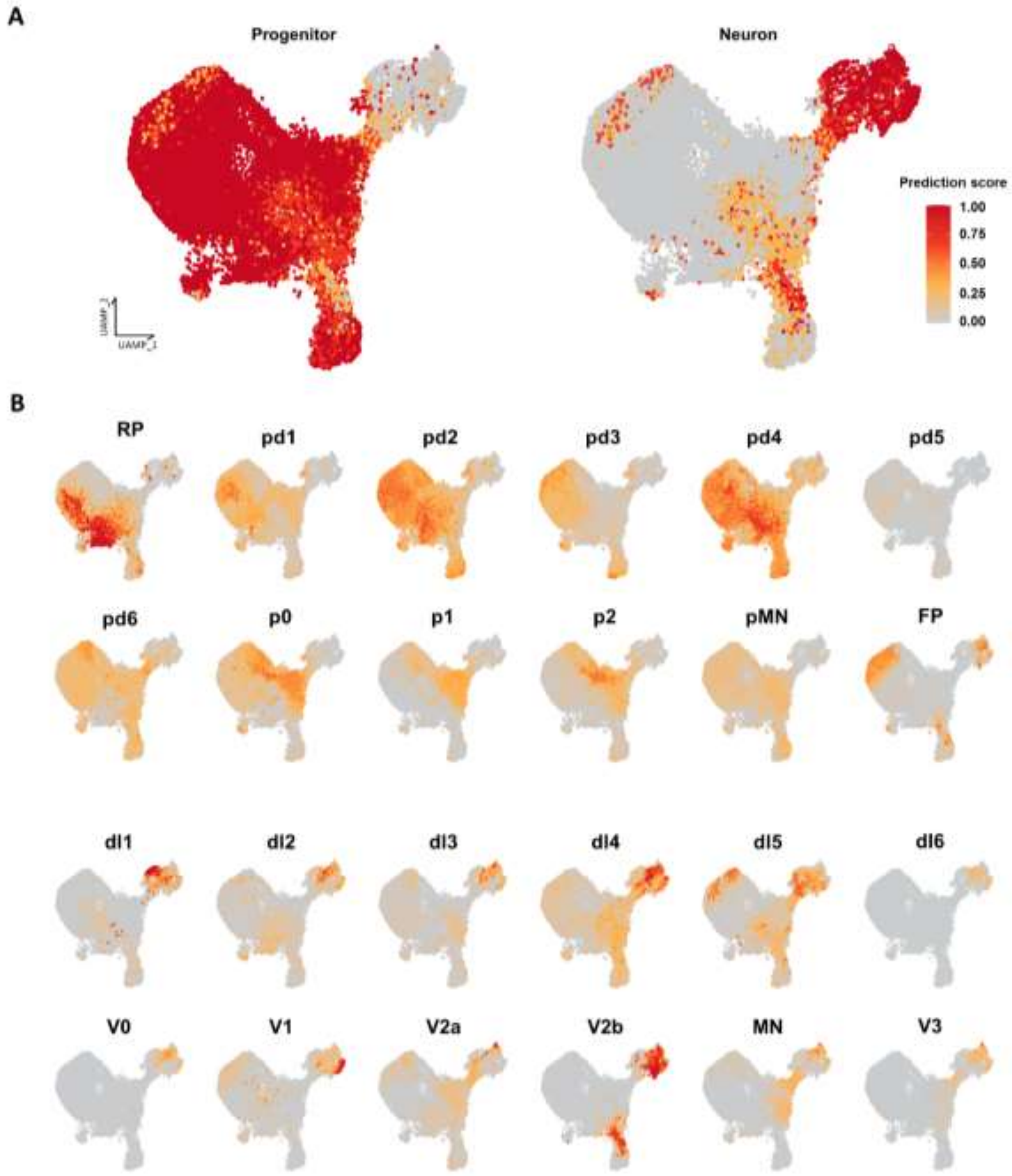

Figure S4. Comparison of pSCOs to developing mouse spinal cord. UMAP plots shows the cell assignment by prediction score analysis from scRNA sequencing clusters from previously published developing mouse spinal cord. Progenitor and neuron clusters (A) and detailed mitotic and postmitotic SC clusters (B).

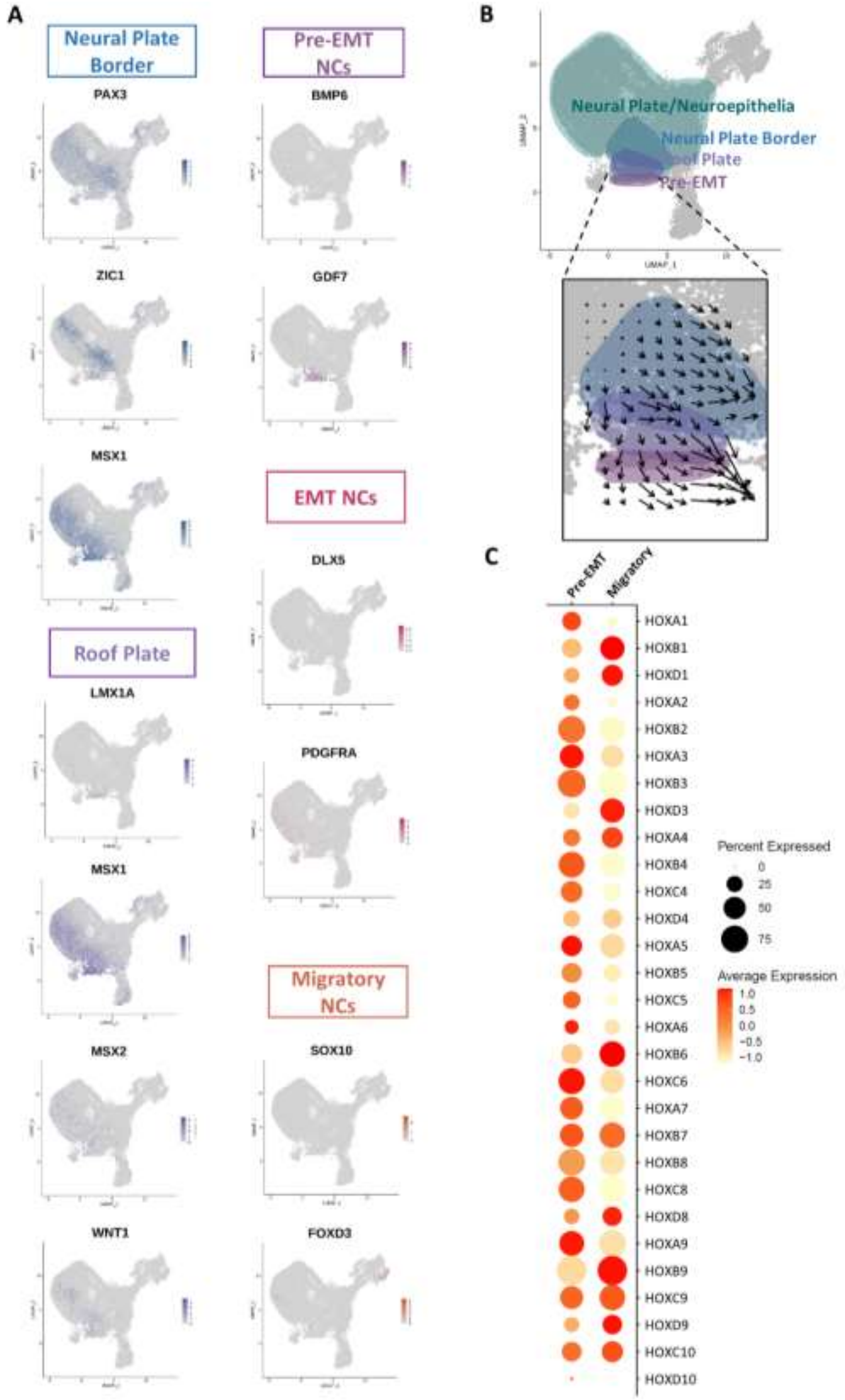

Figure S5. scRNA analysis of neural crest cell (NCC) development. (A) Feature plots of representative marker genes used to assign cluster identities colored based on gene expression

level. (B) Streamline of RNA velocity displays the developmental process of NCCs from neural plate border to pre-EMT state. (C) Dot plots of HOX genes in clusters of pre-EMT and migratory NCCs. The size of each circle indicates the percentage of cells where the indicated gene was detected, and the color intensity reflects the scaled average expression level within cells.

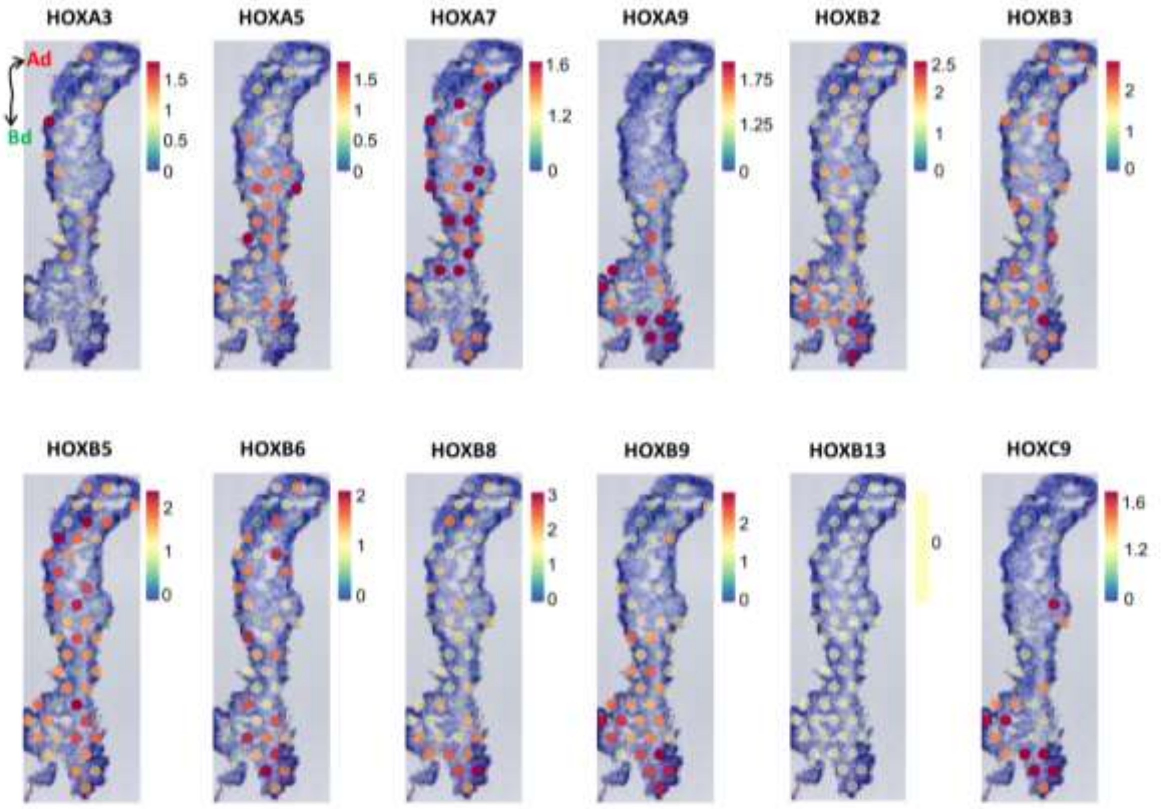

Figure S6. Analysis of HOX gene expression pattern. Mapping of HOX genes in the organoid section. Black double arrowhead is labeled as Ad and Bd indicates the long axis of organoid section.

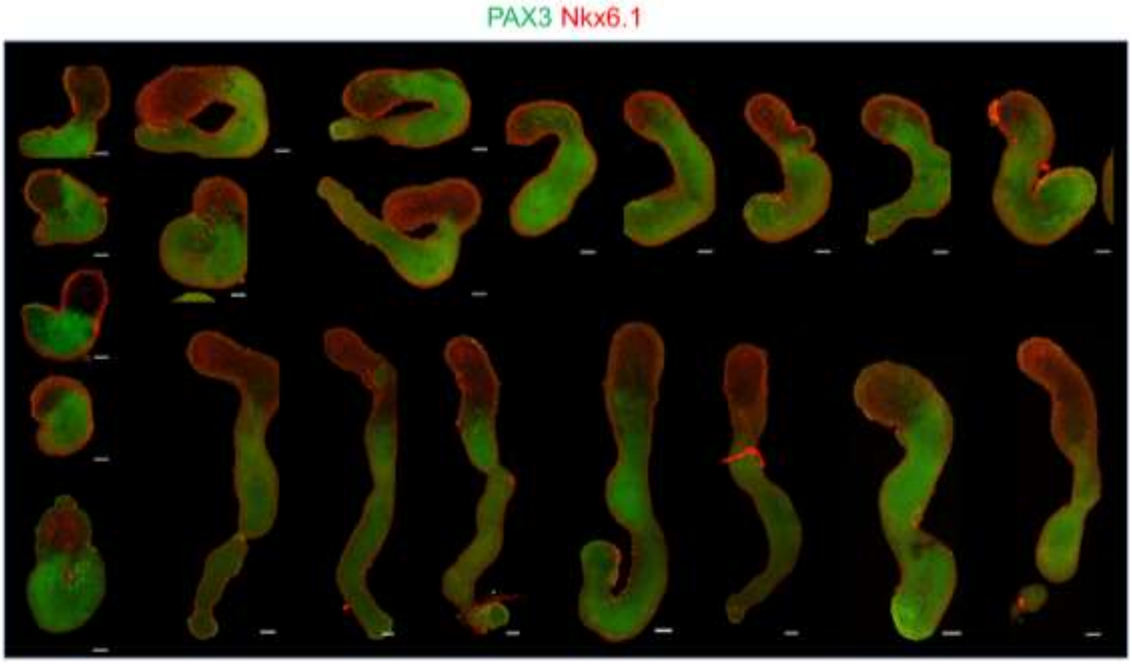

Figure S7. Immunofluorescence images of organoids for quantification of DV spatial patterning in Fig. 5B. Organoids at FGFD6 were costained with anti-Pax3 and anti-Nkx6.1 antibodies. Confocal images were taken in 5-μm steps along the z-axis and three-dimensional renderings were created. Scale bar, 100 μm.

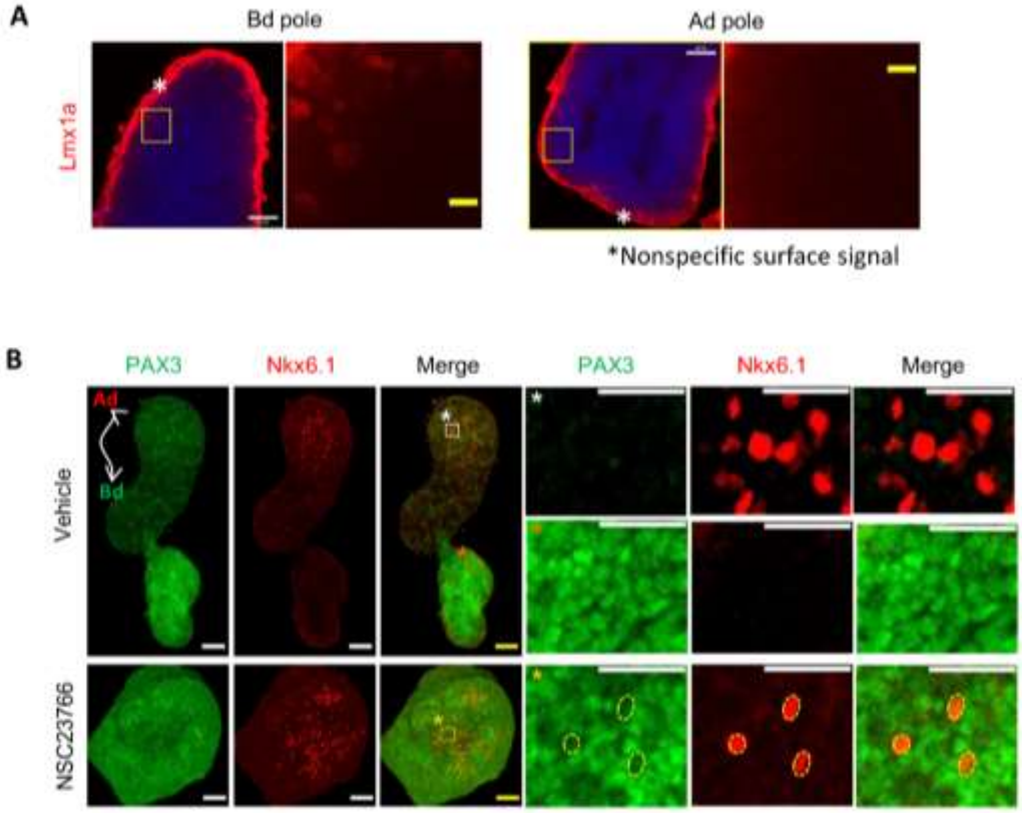

Figure S8. Characterization of DV patterning. (A) Immunofluorescence analysis of a roof plate marker, Lmx1a in the organoid (FGFD6). High-magnification images correspond to the insets. White asterisks indicate the nonspecific surface signal. White scale bar, 100 μm. Yellow scale bar, 10 μm. (B) Effect of Rac inhibitor (NSC23766) on the DV patterning. Organoids were derived from micropatterned colonies treated with NSC23766 or vehicle at SC-D2. High-resolution images from one layer (Scale bar, 30 μm) correspond to insets.

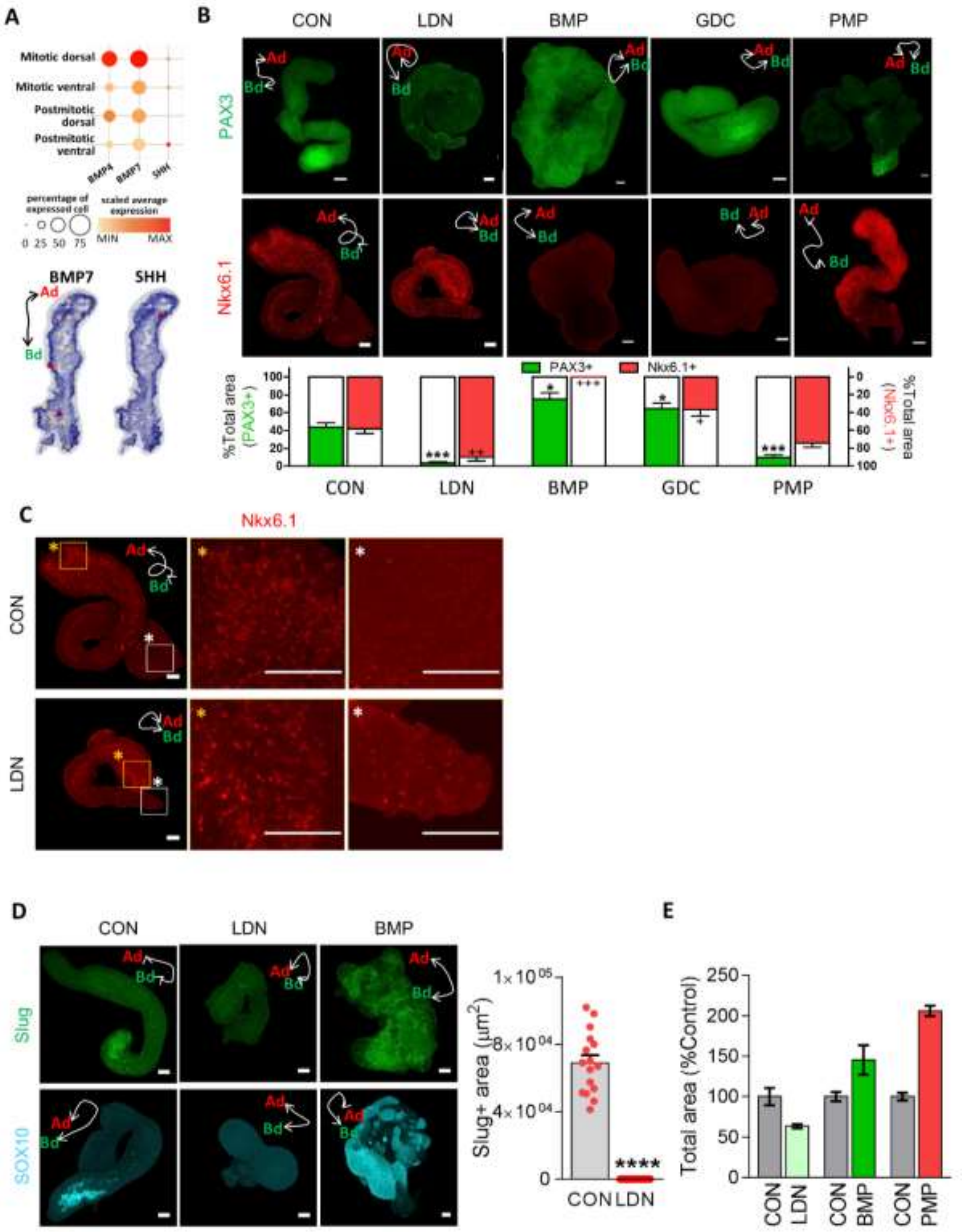

Figure S9. Effect of BMP and Shh signaling activities on DV patterning of organoids. (A) Dot plot showing the expression of BMP and SHH across four main clusters and mapping of BMP and SHH in the organoid section. (B) Effect of BMP signaling inhibitor (LDN), BMP, Shh inhibitor (GDC), and Shh activator (PMP) on DV-like axis organization of pSCOs in the

progenitor stage. Organoids treated with the indicated reagents for 6 days in the presence of bFGF were fixed at FGFD6 and immunostained with the dorsal marker (PAX3) and ventral marker (Nkx6.1). Quantification of PAX3- and Nkx6.1-positive area (% total area) at each condition shown as mean ± SEM (n = 7~11 per group). Statistical significance was tested with One-way of analysis of variance (ANOVA) with Tukey's post hoc test. *, +: $p<0.05$, ++: $p<0.01$, ***, +++: $p<0.001$. (C) Effect of BMP inhibitor (LDN) on the Nkx6.1-positive region and the Nkx6.l expression level in organoids (FGFD6). High-resolution images correspond to insets. (D) Effect of BMP signaling inhibitor (LDN) and BMP on the generation of neural crest cells in pSCOs (FGFD6). Quantification of slug-positive area in the organoid shown as mean ± SEM. Statistical significance was tested with unpaired t-test. ****: $p<0.0001$. (E) Effect of BMP inhibitor (LDN), BMP, and Shh activator (PMP) on the growth of organoids (FGFD6). Quantification of the total area in each group shown as mean ± SEM (n = 9~23 per group). White double arrowhead labeled as Ad and Bd indicates the long axis of organoids. Confocal images were taken in 1- or 5-μm steps along the z-axis after fixation and immunostaining with the indicated antibodies and stacked using Z-stack maximum projection. All images are representative examples from at least three independent experiments. Scale bar, 100 μm.

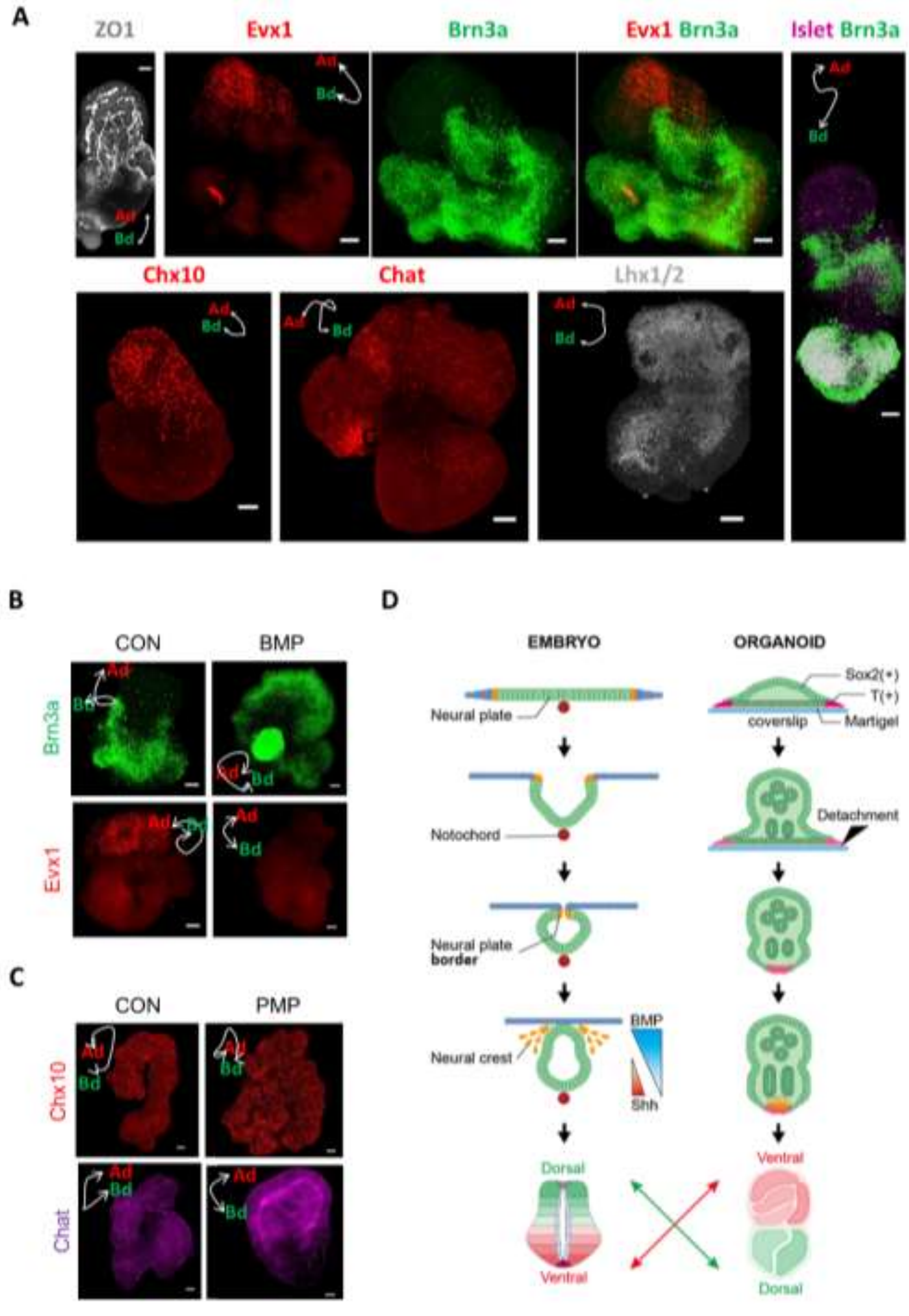

Figure S10. Characterization of spatial dorsoventral axis patterning of pSCOs at postmitotic stage. (A) Immunofluorescence analysis of spinal cord organoids with markers for spinal cord postmitotic neurons. Organoids were fixed at DMD10 (EVX1/Brn3a, Chx10 and Lhx1/2) or at DMD20 (Islet/Brn3a and Chat) and immunostained with the indicated antibodies. (B) Effect of BMP on the DV axis organization of spinal cord organoids in the postmitotic stage.

Organoids treated with the indicated reagents were fixed at DMD20 and immunostained with the dorsal marker (Brn3a) and ventral marker (Evx1). (C) Effect of Shh activator (PMP) on the DV-like axis organization of spinal cord organoids in the postmitotic stage. Organoids treated with the indicated reagents were fixed at DMD20 and immunostained with the ventral markers (Chx10 and Chat). White double arrowhead labeled as Ad and Bd indicates the long axis of organoids. Confocal images were taken in 5-μm steps along the z-axis after fixation and immunostaining with the indicated antibodies, and stacked using Z-stack maximum projection. All images are representative examples from at least three independent experiments. Scale bar, 100 μm. (D) Schematic comparisons of spinal cord DV axis patterning in embryos and polarized DV patterning in organoids. pSCOs recapitulate neural tube development in vivo in many aspects. Neural stem cells at the top part of the organoids become ventral progenitor cells, and cells at the bottom become dorsal cells, leading to dorsal and ventral postmitotic neurons with spatial DV organization reminiscent of the spinal cord DV axis *in vivo*.

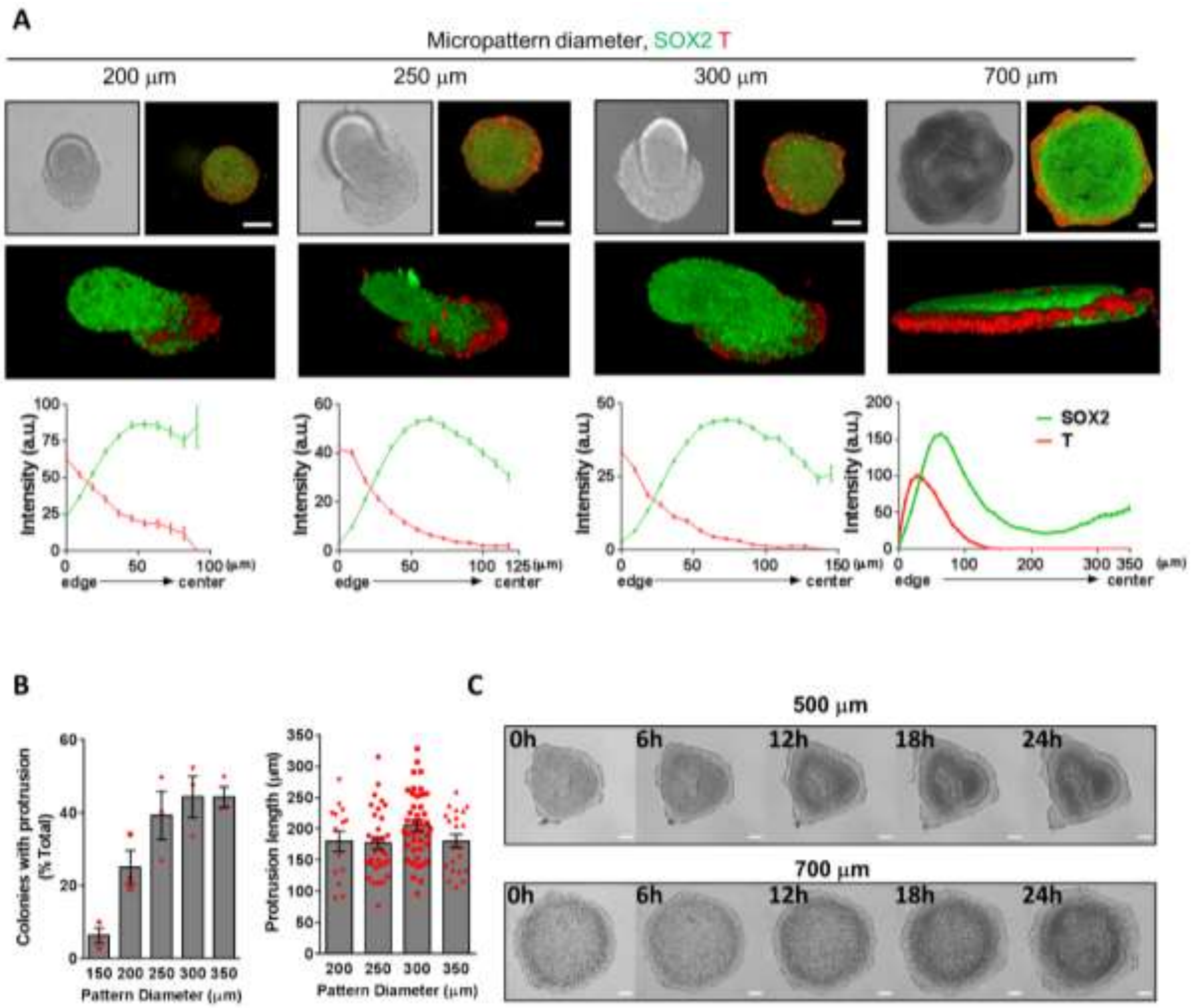

Figure S11. Effects of micropatterned colony size on spatial cell patterning and colony morphogenesis. (A) Immunofluorescence analysis and quantification of colonies grown on micropatterns with the indicated diameters at Day 3 of SB/Chir treatment. Confocal images were taken in 5-μm steps along the z-axis after fixation and immunostaining with the indicated antibodies, and three-dimensional renderings were created. Quantification of fluorescent intensities at each position shown as mean ± SEM (n = total 7005 data points from 24 images at 200 μm, total 19062 data points from 31 images at 250 μm, total 31108 data points from 39 images at 300 μm, total 38138 data points from 6 images at 700 μm). (B) Percentage of colonies with the protrusion, and length of the protrusion were quantified at

SCD3. Data shown as mean ± SEM. (C) Bright field images of colony morphogenesis developing during the 3rd day of SB/Chir treatment.

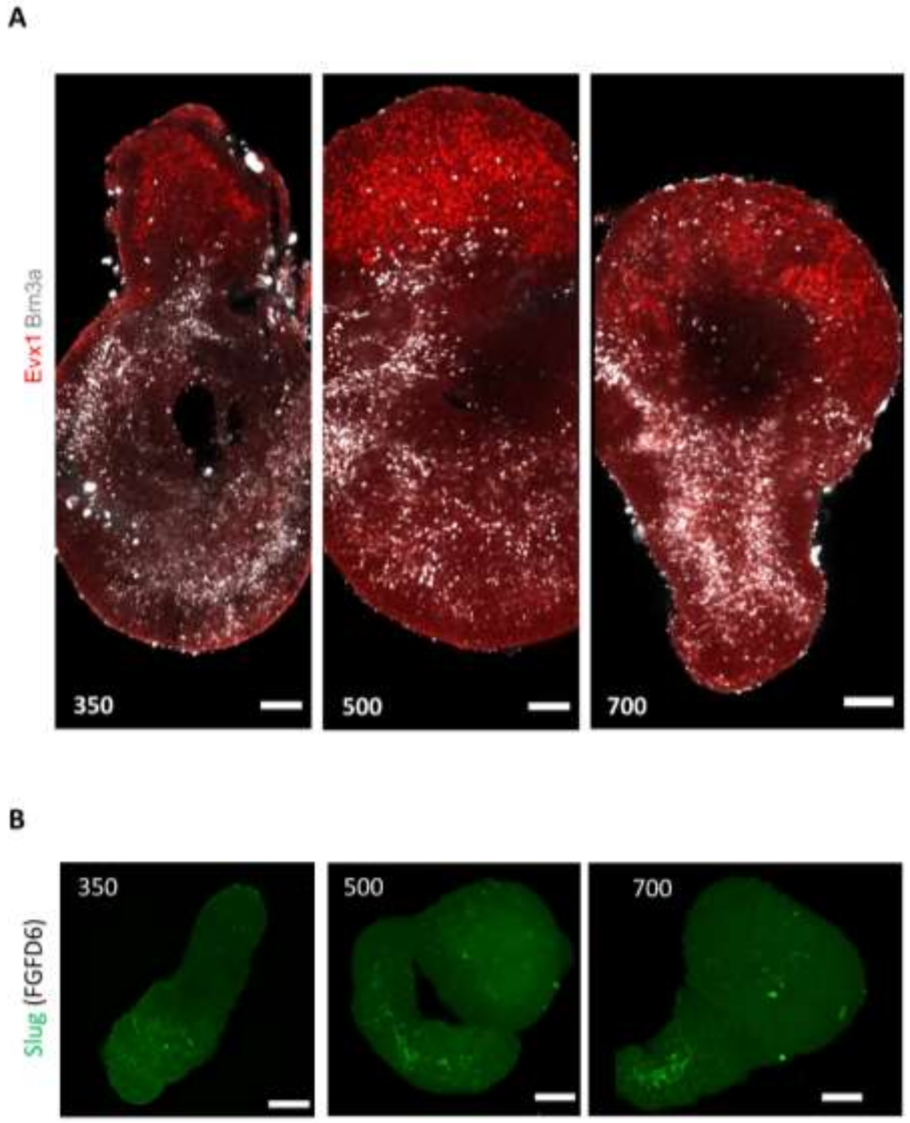

Figure S12. Characterization of pSCOs derived from colonies with different diameters. Organoids derived from 350-, 500- and 700 μm were stained with spinal cord postmitotic neuronal markers (A, DMD20) and NC marker (B, FGFD6). Confocal images were taken in 5-μm steps along the z-axis after fixation and immunostaining with the indicated antibodies, and stacked using Z-stack maximum projection. All images are representative examples from at least three independent experiments. Scale bar, 100 μm.

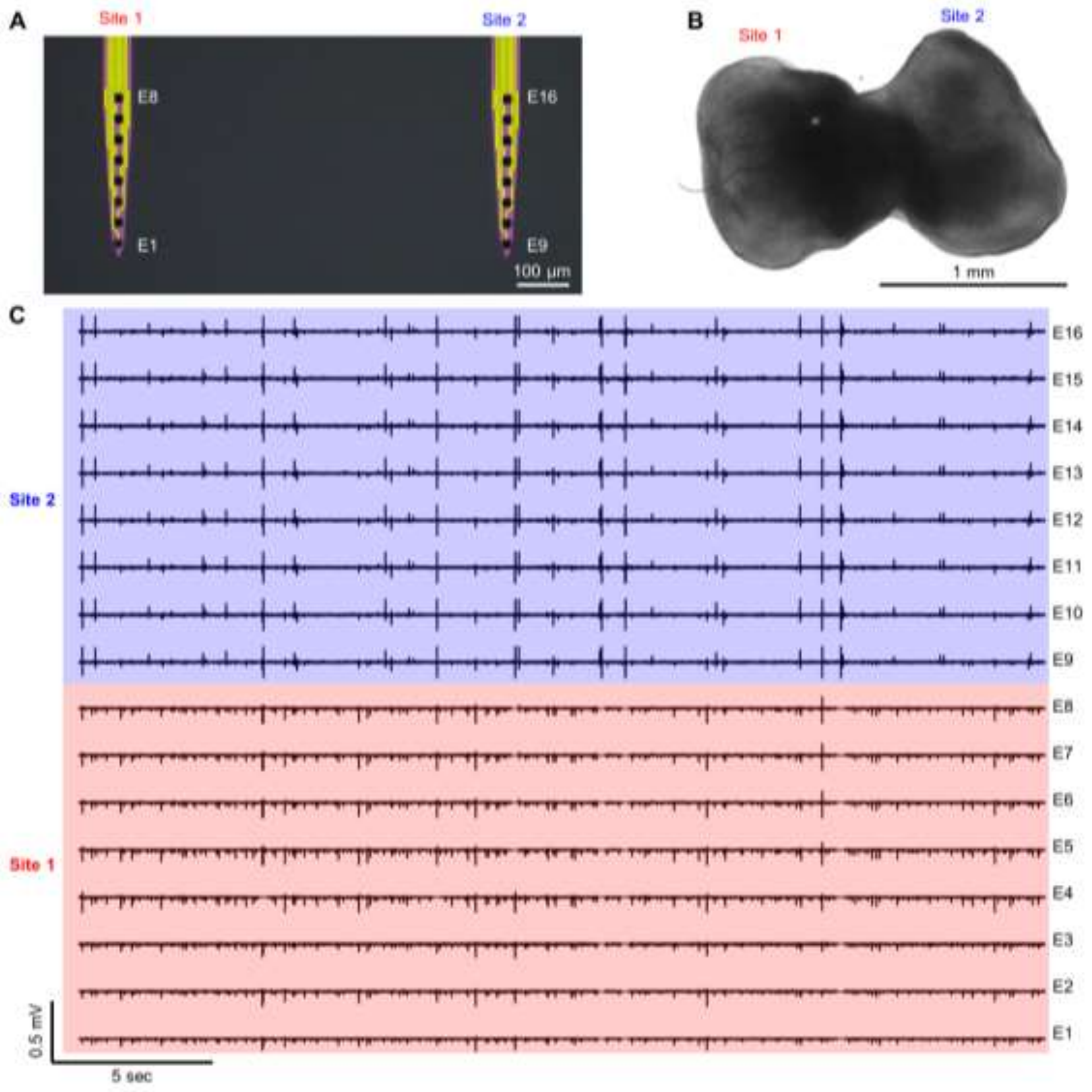

Figure S13. The neural signal recording from pSCOs using a 2-shank neural probe. (A) The representative optical image of the 2-shank neural probe with 16 black Pt electrodes. Electrodes 1-8 were inserted into site 1 of the pSCO, and electrodes 9-16 were inserted into site 2 of the pSCO. (B) The representative optical image of pSCOs (DMD73). (C) The transient plot of neural signals recorded from sites 1 and 2 in the pSCO.

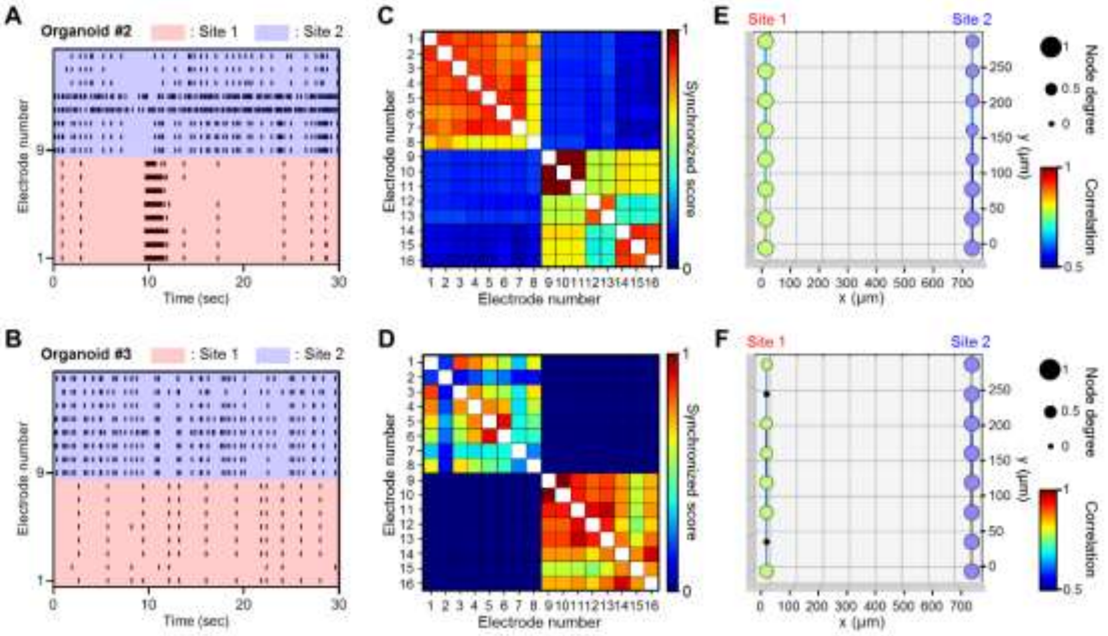

Figure S14. Detailed results of neural signal recording from other pSCOs. (A, B) Representative raster plot of neural signals recorded from sites 1 and 2 in organoids 2 and 3 (DMD73). Specifically, electrodes 1-8 were inserted into site 1 of the pSCOs, and electrodes 9-16 were inserted into site 2 of the pSCOs. (C, D) The cross-correlation matrix showing synchronized scores between electrodes in organoids 2 and 3. (E, F) The 2D network map displaying connectivity between electrodes in organoids 2 and 3. Node color represents the network connection among electrodes. Node degree reflects the number of electrodes connected to each electrode. The line color indicates the correlation between the electrodes.

Table S1. Key resources

REAGENT or RESOURCE	SOURCE	IDENTIFIER
Antibodies		
Rabbit polyclonal anti-Sox2	Millipore	Cat# AB5603; RRID: AB_2286686
Mouse monoclonal anti-Sox2	Santa Cruz Biotechnology	Cat# sc-365823; RRID: AB_10842165
Goat polyclonal anti-Brachyury	R&D Systems	Cat# AF2085; RRID: AB_2200235
Rabbit polyclonal anti-beta-Catenin	Sigma-Aldrich	Cat# C2206; RRID: AB_476831
Rabbit polyclonal anti-ZO-1	Thermo Fisher Scientific	Cat# 61-7300; RRID: AB_2533938
Rabbit monoclonal anti-CDX2	Abcam	Cat# ab76541; RRID: AB_1523334
Goat polyclonal anti-Doublecortin(DCX)	Santa Cruz Biotechnology	Cat# sc-8066; RRID: AB_2088494
Goat polyclonal anti-Nkx6.1 (N-15)	Santa Cruz Biotechnology	Cat# sc-15027; RRID: AB_650286
Mouse monoclonal anti-Pax3	R&D Systems	Cat# 274212; RRID: AB_2159398
Mouse monoclonal anti-Pax6	DSHB	Cat# pax6; RRID: AB_528427
Rabbit monoclonal anti-Olig3	Abcam	Cat# Ab129197; RRID: AB_11142825
Mouse monoclonal anti-Pax7	DSHB	Cat# pax7; RRID: AB_528428
Rabbit monoclonal anti-Slug (C19G7)	Cell Signaling Technology	Cat# 9585; RRID: AB_2239535
Mouse monoclonal anti-Sox10 (A-2)	Santa Cruz Biotechnology	Cat# sc-365692; RRID: AB_10844002
Mouse monoclonal anti-Evx1/2	DSHB	Cat# 99.1-3a2; RRID: AB_528231
Goat polyclonal anti-Brn3a (C-20)	Santa Cruz Biotechnology	Cat# sc-31984; RRID: AB_2167511
Rabbit polyclonal anti-Islet1	Abcam	Cat# Ab20670; RRID: AB_881306
Sheep polyclonal anti-Chx10 (N-terminus)	Millipore	Cat# AB9016; RRID: AB_2216009
Goat polyclonal anti-Chat	Millipore	Cat# AB144P; RRID: AB_2079751
Mouse monoclonal anti-Lim 1+2 / LhxV5	DSHB	Cat# 4F2; RRID: AB_531784
Rabbit polyclonal anti-Peripherin	Abcam	Cat# ab4666; RRID: AB_449340
Mouse monoclonal anti-N-Cadherin	BD Biosciences	Cat# 610921; RRID: AB_398236
Hoechst33342	Invitrogen	Cat# H3570; CAS: 23491-52-3
Donkey anti-Mouse IgG (H+L) Antibody, Alexa Fluor 488	Thermo Fisher Scientific	Cat# A21202; RRID: AB_141607
Donkey anti-Rabbit IgG (H+L) Antibody, Alexa Fluor 488	Thermo Fisher Scientific	Cat# A21206; RRID: AB_2535792
Donkey anti-Goat IgG (H+L) Antibody, Alexa Fluor 488	Thermo Fisher Scientific	Cat# A11055; RRID: AB_2534102
Donkey anti-Sheep IgG (H+L) Antibody, Alexa Fluor 488	Thermo Fisher Scientific	Cat# A21448; RRID: AB_2534082

Donkey anti-Mouse IgG (H+L) antibody, Cy3	Jackson ImmunoResearch	Cat# 715-165-151; RRID: AB_2315777
Donkey anti-Rabbit IgG (H+L) antibody, Cy3	Jackson ImmunoResearch	Cat# 711-165-152; RRID: AB_2307443
Donkey anti-Goat IgG (H+L) antibody, Cy3	Jackson ImmunoResearch	Cat# 705-165-147; RRID: AB_2307351
Donkey anti-Mouse IgG (H+L) antibody, Alexa Fluor 647	Jackson ImmunoResearch	Cat# 715-606-150; RRID: AB_2340865
Donkey anti-Rabbit IgG (H+L) antibody, Alexa Fluor 647	Jackson ImmunoResearch	Cat# 711-605-152; RRID: AB_2492288
Donkey anti-Goat IgG (H+L) antibody, Alexa Fluor 647	Thermo Fisher Scientific	Cat# A21447; RRID: AB_2535864
Chemicals, Peptides, and Recombinant Proteins		
SB 431542	TOCRIS	Cat# 1614; CAS: 301836-41-9
CHIR 99021	Sigma-Aldrich	Cat# SML1046; CAS: 252917-06-9
LDN-193189	Stemgent	Cat# 04-0074; CAS: 1062368-24-4
Human bFGF	R&D Systems	Cat# 233-FB; GenPept: P09038
Human BMP2	PEPROTECH	Cat# 120-02; GenPept P12643
Purmorphamine	Sigma-Aldrich	Cat# 540220; CAS: 483367-10-8
Matrigel hESC-Qualified Matrix	Corning	Cat# 354277
Cell Recovery Solution	Corning	Cat# 354253
mTeSR1	STEMCELL Technologies	Cat# 85850
ReLeSR	STEMCELL Technologies	Cat# 05872
DMEM/F-12	Gibco	Cat# 11320033
N2	Gibco	Cat# 17502048
B27	Gibco	Cat# 17504044
Nonessential amino acids (NEAA)	Gibco	Cat# 11140050
penicillin/streptomycin (P/S)	Gibco	Cat# 15140122
β-mercaptoethanol	Gibco	Cat# 21985023
HBSS	Gibco	Cat# 14175095
Experimental Models: Cell Lines		
Human: Passage 30 H9 ES cells	WiCell	WA09; WAe009-A
Software and Algorithms		
ImageJ-Fiji	Schneider et al., 2012	https://imagej.net/Fiji
LasX	Leica	https://www.leica-microsystems.com/products/microscope-software/p/leica-las-x-ls/
Prism5	GraphPad	https://www.graphpad.com/
MATLAB	MathWorks	https://kr.mathworks.com/products/matlab.html
SigmaPlot 12	SYSTAT	https://systatsoftware.com/downloads/download-sigmaplot/

Supplementary data 1. Top ten markers for 6 main clusters (Figure 3A and 3B related)

cluster	gene	p_val	avg_log2FC	pct.1	pct.2	p_val_adj
Neural Progenitor	CDX4	0	2.807732	0.738	0.163	0
Neural Progenitor	L1TD1	0	2.119606	0.611	0.121	0
Neural Progenitor	LIN28A	0	2.106677	0.724	0.266	0
Neural Progenitor	HOXA-AS3	0	1.843626	0.608	0.163	0
Neural Progenitor	HIST1H1A	0	1.843369	0.541	0.187	0
Neural Progenitor	HIST1H4C	0	1.831013	0.835	0.675	0
Neural Progenitor	S100A11	0	1.774463	0.733	0.305	0
Neural Progenitor	KRT18	0	1.76902	0.68	0.235	0
Neural Progenitor	NKX1-2	0	1.768218	0.503	0.049	0
Neural Progenitor	CDX2	0	1.741795	0.542	0.058	0
Mitotic Dorsal	MSX1	0	1.892864	0.713	0.375	0
Mitotic Dorsal	TPBG	0	1.819397	0.472	0.23	0
Mitotic Dorsal	ZIC1	0	1.60083	0.422	0.073	0
Mitotic Dorsal	LINC01933	0	1.32294	0.327	0.069	0
Mitotic Dorsal	COL1A2	0	1.249502	0.449	0.236	0
Mitotic Dorsal	TPPP3	0	1.150476	0.494	0.221	0
Mitotic Dorsal	DDIT4	1.31E-259	1.155827	0.71	0.528	3.23E-255
Mitotic Dorsal	RARRES2	5.12E-241	1.509504	0.42	0.24	1.26E-236
Mitotic Dorsal	SLC2A1	2.63E-131	1.210929	0.733	0.636	6.46E-127
Mitotic Dorsal	ID1	3.84E-23	2.2723	0.66	0.646	9.45E-19
Mitotic Ventral	HES5	0	2.783612	0.887	0.305	0
Mitotic Ventral	PLP1	0	2.691826	0.872	0.335	0
Mitotic Ventral	FGFBP3	0	2.562588	0.875	0.621	0
Mitotic Ventral	SFRP2	0	2.451446	0.944	0.708	0
Mitotic Ventral	SFRP1	0	2.382564	0.937	0.64	0
Mitotic Ventral	VIM	0	2.284012	1	0.984	0
Mitotic Ventral	PAX6	0	2.212155	0.904	0.269	0
Mitotic Ventral	TTYH1	0	2.201855	0.966	0.568	0
Mitotic Ventral	PANTR1	0	2.101576	0.789	0.195	0
Mitotic Ventral	SOX3	0	1.974342	0.923	0.524	0
Postmitotic Dorsal	CRABP1	0	4.063777	0.833	0.426	0
Postmitotic Dorsal	DLL3	0	3.060043	0.57	0.143	0
Postmitotic Dorsal	TUBB3	0	2.712647	0.996	0.894	0
Postmitotic Dorsal	TAGLN3	0	2.705269	0.832	0.374	0

Postmitotic Dorsal	IGFBPL1	0	2.620983	0.807	0.376	0
Postmitotic Dorsal	MIAT	0	2.459304	0.928	0.443	0
Postmitotic Dorsal	SOX4	0	2.458539	0.992	0.893	0
Postmitotic Dorsal	POU2F2	0	2.397806	0.475	0.076	0
Postmitotic Dorsal	HES6	5.81E-269	5.089067	0.815	0.58	1.43E-264
Postmitotic Dorsal	RGS16	9.94E-127	2.677158	0.473	0.242	2.45E-122
Postmitotic Ventral	PCP4	0	5.064343	0.563	0.053	0
Postmitotic Ventral	STMN2	0	4.594193	0.901	0.362	0
Postmitotic Ventral	STMN4	0	3.729452	0.76	0.094	0
Postmitotic Ventral	DCX	0	3.559094	0.881	0.273	0
Postmitotic Ventral	CRABP1	0	3.328146	0.832	0.411	0
Postmitotic Ventral	KIF5C	0	3.265042	0.926	0.607	0
Postmitotic Ventral	TUBB3	0	3.155997	0.952	0.894	0
Postmitotic Ventral	NOVA1	0	3.10056	0.777	0.411	0
Postmitotic Ventral	LAMP5	0	3.0924	0.818	0.399	0
Postmitotic Ventral	CDKN1C	0	3.078123	0.908	0.57	0
early mesoderm	RSPO3	0	2.322555	0.426	0.018	0
early mesoderm	DKK1	0	2.313347	0.429	0.009	0
early mesoderm	MSGN1	0	2.25307	0.462	0.003	0
early mesoderm	APLNR	0	1.927947	0.472	0.027	0
early mesoderm	FOXC2	0	1.907757	0.429	0.009	0
early mesoderm	CITED1	2.43E-249	2.49826	0.518	0.096	5.99E-245
early mesoderm	KRT19	1.61E-152	2.546227	0.65	0.215	3.95E-148
early mesoderm	MEST	3.38E-141	2.751874	0.954	0.715	8.32E-137
early mesoderm	FABP5	3.81E-99	2.00874	0.971	0.83	9.39E-95
early mesoderm	NTS	2.31E-56	2.085785	0.317	0.108	5.69E-52

Supplementary data 2. Top ten markers for sub-clustered dorsal and ventral clusters (Figure 3C related)

cluster	gene	p_val	avg_log2FC	pct.1	pct.2	p_val_adj
NP1	CDX4	0	1.990479	0.869	0.182	0
NP1	HIST1H4C	0	1.700581	0.947	0.648	0
NP1	L1TD1	0	1.627712	0.744	0.127	0
NP1	LIN28A	0	1.548733	0.869	0.263	0
NP1	NKX1-2	0	1.433722	0.623	0.056	0
NP1	CENPF	0	1.390822	0.988	0.416	0
NP1	CDX2	0	1.380813	0.667	0.067	0
NP1	DSP	0	1.329194	0.86	0.371	0
NP1	MKI67	0	1.328998	0.901	0.248	0
NP1	HOXA-AS3	0	1.306151	0.742	0.163	0
NP1	TPX2	0	1.272021	0.966	0.413	0
NP1	KRT18	0	1.265221	0.823	0.231	0
NP1	MLEC	0	1.264357	0.973	0.687	0
NP1	ASPM	0	1.21552	0.826	0.213	0
NP1	DUSP6	0	1.214704	0.753	0.177	0
NP1	HIST1H1A	0	1.181057	0.653	0.185	0
NP1	FST	0	1.096547	0.714	0.319	0
NP1	S100A11	0	1.063703	0.874	0.3	0
NP1	HIST1H1B	0	1.042062	0.565	0.114	0
NP1	SMC4	0	1.024796	0.89	0.308	0
NP2	RNMT	5.88E-240	1.368323	0.227	0.703	1.45E-235
NP2	PRPF4B	1.67E-217	1.281486	0.207	0.643	4.10E-213
NP2	UPF2	2.46E-188	1.462153	0.179	0.563	6.06E-184
NP2	DDX18	8.74E-131	1.330921	0.331	0.802	2.15E-126
NP2	EIF5B	3.30E-108	1.41777	0.347	0.801	8.13E-104
NP2	TOP1	1.90E-95	1.277632	0.368	0.825	4.68E-91
NP2	SPC25	7.78E-78	1.252398	0.13	0.333	1.92E-73
NP2	DDX46	5.90E-77	1.364422	0.364	0.787	1.45E-72
NP2	GMNN	1.70E-76	1.295485	0.249	0.544	4.17E-72
NP2	CDKN3	2.96E-66	1.293753	0.205	0.445	7.28E-62
NP2	DLGAP5	1.86E-52	1.338556	0.193	0.395	4.59E-48
NP2	CDK1	3.60E-44	1.257228	0.171	0.342	8.87E-40
NP2	HIST1H1A	1.83E-42	1.370219	0.172	0.34	4.51E-38
NP2	TUBA1C	5.79E-40	1.312116	0.313	0.59	1.43E-35
NP2	S100A11	1.21E-35	1.458237	0.265	0.492	2.99E-31

NP2	AURKA	1.53E-33	1.3739	0.162	0.308	3.78E-29
NP2	KPNA2	6.33E-25	1.407962	0.431	0.802	1.56E-20
NP2	NUSAP1	1.13E-18	1.497676	0.293	0.489	2.78E-14
NP2	CCNB1	9.07E-10	1.422658	0.332	0.516	2.23E-05
NP2	PCLAF	1.18E-09	1.425897	0.359	0.572	2.90E-05
RP	MAL	0	3.902565	0.785	0.031	0
RP	TPBG	0	3.563016	0.9	0.248	0
RP	MSX1	0	3.511705	0.951	0.41	0
RP	COL1A2	0	2.66281	0.748	0.254	0
RP	GDF7	0	2.658776	0.62	0.013	0
RP	APCDD1	0	2.605657	0.644	0.139	0
RP	NFIA	0	2.537243	0.684	0.06	0
RP	MAFB	0	2.504016	0.462	0.062	0
RP	ADCY2	0	2.433383	0.708	0.058	0
RP	DMD	0	2.413444	0.76	0.202	0
RP	CCDC80	0	2.2873	0.667	0.214	0
RP	ALDH1A2	0	1.97683	0.652	0.02	0
RP	DDIT4	3.99E-293	2.467564	0.944	0.544	9.82E-289
RP	ID1	2.11E-271	3.929997	0.891	0.642	5.19E-267
RP	COL2A1	6.14E-268	2.150199	0.821	0.437	1.51E-263
RP	RARRES2	7.02E-264	2.478229	0.676	0.255	1.73E-259
RP	ID2	8.68E-248	2.218481	0.849	0.494	2.14E-243
RP	CNTNAP2	3.82E-215	2.163848	0.686	0.332	9.40E-211
RP	ATP1B1	6.69E-195	2.009734	0.785	0.464	1.65E-190
RP	SLC2A1	1.41E-135	2.598345	0.817	0.645	3.47E-131
dp2	OLIG3	1.69E-245	1.457284	0.639	0.18	4.15E-241
dp2	ZIC2	1.39E-152	0.995605	0.882	0.436	3.42E-148
dp2	CDX4	1.16E-151	1.052956	0.855	0.377	2.85E-147
dp2	MSX1	1.62E-133	1.290184	0.785	0.417	3.98E-129
dp2	WNT4	1.40E-112	1.144297	0.349	0.1	3.44E-108
dp2	HOXB-AS3	9.08E-108	1.154767	0.975	0.776	2.24E-103
dp2	GREM1	6.03E-98	0.762008	0.287	0.078	1.48E-93
dp2	COL14A1	1.80E-96	0.853946	0.605	0.292	4.44E-92
dp2	MCM4	2.08E-94	0.84165	0.74	0.417	5.12E-90

dp2	GINS2	7.22E-90	0.785379	0.895	0.601	1.78E-85
dp2	DTL	3.73E-89	0.815254	0.532	0.247	9.19E-85
dp2	S100A10	4.29E-87	1.215883	0.759	0.48	1.06E-82
dp2	MCM5	1.04E-86	0.755188	0.626	0.328	2.57E-82
dp2	MDK	2.49E-79	0.866648	1	0.893	6.12E-75
dp2	TPBG	1.90E-77	0.995252	0.529	0.26	4.67E-73
dp2	MCM6	1.45E-76	0.815497	0.627	0.349	3.57E-72
dp2	PCNA	1.28E-75	0.76208	0.784	0.521	3.14E-71
dp2	GDF10	9.53E-73	0.734077	0.33	0.119	2.34E-68
dp2	DRAXIN	1.47E-67	0.899554	0.651	0.39	3.63E-63
dp2	SPARCL1	1.43E-62	0.753077	0.364	0.151	3.52E-58
dp4	ZIC1	0	1.883289	0.496	0.083	0
dp4	LINC01933	0	1.553912	0.369	0.078	0
dp4	PTN	0	1.462278	0.916	0.707	0
dp4	CLEC3A	0	1.374983	0.265	0.04	0
dp4	PAX3	0	1.228405	0.601	0.235	0
dp4	SCUBE2	0	1.037233	0.493	0.232	0
dp4	ZIC4	0	0.93792	0.252	0.04	0
dp4	HOXA9	0	0.892206	0.629	0.336	0
dp4	WNT4	0	0.79299	0.287	0.085	0
dp4	GAS1	1.21E-261	0.864101	0.641	0.393	2.97E-257
dp4	TMEM47	8.11E-260	0.873386	0.81	0.636	2.00E-255
dp4	ARL4A	1.80E-252	0.786057	0.781	0.596	4.43E-248
dp4	NEDD9	4.37E-249	0.792615	0.442	0.219	1.08E-244
dp4	TPPP3	2.08E-194	0.905008	0.452	0.239	5.12E-190
dp4	EDNRB	7.96E-190	0.763192	0.414	0.212	1.96E-185
dp4	GPC3	2.18E-178	0.73601	0.787	0.616	5.37E-174
dp4	METRN	8.57E-171	0.714081	0.892	0.783	2.11E-166
dp4	NPPC	1.20E-117	0.729147	0.386	0.225	2.95E-113
dp4	CALCB	2.20E-111	1.380658	0.31	0.168	5.41E-107
dp4	RARRES2	1.06E-104	0.9044	0.393	0.252	2.62E-100
MV	HES5	0	2.783612	0.887	0.305	0
MV	PLP1	0	2.691826	0.872	0.335	0
MV	FGFBP3	0	2.562588	0.875	0.621	0
MV	SFRP2	0	2.451446	0.944	0.708	0
MV	SFRP1	0	2.382564	0.937	0.64	0

MV	VIM	0	2.284012	1	0.984	0
MV	PAX6	0	2.212155	0.904	0.269	0
MV	TTYH1	0	2.201855	0.966	0.568	0
MV	PANTR1	0	2.101576	0.789	0.195	0
MV	SOX3	0	1.974342	0.923	0.524	0
MV	CD99	0	1.908768	0.955	0.675	0
MV	CRYGD	0	1.891707	0.636	0.2	0
MV	IGDCC3	0	1.797479	0.979	0.829	0
MV	AL139246.5	0	1.663613	0.603	0.156	0
MV	PCDH17	0	1.586589	0.683	0.18	0
MV	NRARP	0	1.462269	0.755	0.324	0
MV	FZD7	0	1.448698	0.783	0.377	0
MV	POU3F2	0	1.392956	0.813	0.352	0
MV	LFNG	0	1.371783	0.617	0.197	0
MV	JAG1	2.22E-234	1.432355	0.4	0.268	5.47E-230
dl1	HES6	0	6.050534	0.976	0.581	0
dl1	DLL3	0	3.596055	0.711	0.149	0
dl1	TMEM176A	0	2.390059	0.756	0.11	0
dl1	LINC01933	0	2.245654	0.653	0.098	0
dl1	ATOH1	0	2.225363	0.444	0.004	0
dl1	SSTR2	1.04E-289	2.712007	0.504	0.089	2.55E-285
dl1	RBP1	4.94E-237	2.297131	0.991	0.838	1.22E-232
dl1	CLEC18A	3.00E-233	2.915771	0.494	0.102	7.39E-229
dl1	IGFBPL1	8.56E-233	2.928936	0.853	0.383	2.11E-228
dl1	GADD45G	1.01E-217	2.433386	0.52	0.113	2.50E-213
dl1	PCBP4	2.76E-199	2.060076	0.88	0.439	6.79E-195
dl1	TUBB3	2.97E-198	2.284728	0.993	0.896	7.32E-194
dl1	TAGLN3	2.24E-195	2.404959	0.824	0.382	5.51E-191
dl1	RGS16	1.77E-178	3.399587	0.653	0.243	4.36E-174
dl1	SOX4	8.12E-163	2.310159	0.988	0.894	2.00E-158
dl1	POU2F2	5.69E-152	2.139588	0.386	0.084	1.40E-147
dl1	CRABP1	1.37E-135	4.370732	0.756	0.434	3.38E-131
dl1	RARRES2	2.12E-113	3.160593	0.581	0.261	5.22E-109
dl1	DRAXIN	1.66E-106	2.077903	0.692	0.39	4.10E-102

dl1	ID1	1.42E-09	3.245647	0.624	0.649	3.51E-05
dl4	STMN2	0	2.93789	0.927	0.39	0
dl4	MIAT	0	2.872382	0.968	0.45	0
dl4	STMN4	0	2.826099	0.768	0.128	0
dl4	DCX	0	2.732416	0.965	0.303	0
dl4	EEF1A2	0	2.654135	0.689	0.157	0
dl4	ELAVL3	0	2.611833	0.863	0.159	0
dl4	POU2F2	0	2.430609	0.558	0.081	0
dl4	RAB3A	0	2.404438	0.718	0.113	0
dl4	CNTN2	0	2.367893	0.479	0.036	0
dl4	TUBB3	1.30E-301	2.822208	0.998	0.896	3.20E-297
dl4	CDKN1C	7.92E-290	2.634929	0.984	0.586	1.95E-285
dl4	CRABP1	7.47E-262	2.834311	0.906	0.431	1.84E-257
dl4	SOX4	5.17E-243	2.387782	0.995	0.894	1.27E-238
dl4	TAGLN3	1.34E-239	2.720524	0.839	0.382	3.29E-235
dl4	ENO2	4.62E-239	2.264803	0.885	0.44	1.14E-234
dl4	MAP2	1.12E-237	2.296795	0.863	0.422	2.77E-233
dl4	SH3BGRL3	3.61E-231	2.294452	0.955	0.722	8.90E-227
dl4	RBP1	1.23E-211	2.15498	0.977	0.838	3.04E-207
dl4	NEFL	1.76E-138	2.360419	0.671	0.324	4.33E-134
dl4	NEFM	2.49E-119	2.358938	0.702	0.355	6.12E-115
V0	PCP4	0	5.265286	0.683	0.067	0
V0	STMN2	0	4.110381	1	0.378	0
V0	STMN4	0	3.576226	0.908	0.112	0
V0	DCX	0	3.567428	0.998	0.29	0
V0	NHLH2	0	3.192467	0.793	0.051	0
V0	TUBB3	0	3.12406	1	0.894	0
V0	CDKN1C	0	3.049377	0.991	0.579	0
V0	RBP1	0	2.959979	0.994	0.835	0
V0	CRABP1	0	2.942614	0.953	0.421	0
V0	GNG3	0	2.721088	0.752	0.1	0
V0	MLLT11	0	2.717096	0.976	0.628	0
V0	LAMP5	0	2.670805	0.906	0.411	0
V0	ELAVL2	0	2.668453	0.908	0.174	0
V0	POU2F2	0	2.6643	0.696	0.068	0
V0	KIF5C	0	2.658812	0.978	0.617	0

V0	SOX4	0	2.555398	0.999	0.892	0
V0	RND3	0	2.554702	0.776	0.213	0
V0	ELAVL3	0	2.543825	0.882	0.147	0
V0	PDLIM1	0	2.524108	0.722	0.371	0
V0	NEFL	0	2.431369	0.735	0.315	0
V1	STMN2	0	4.032067	1	0.391	0
V1	EN1	0	4.015014	0.866	0.006	0
V1	CRABP1	0	3.709474	1	0.432	0
V1	FOXD3-AS1	0	3.56705	0.83	0.016	0
V1	LAMP5	0	3.48595	0.984	0.42	0
V1	STMN4	0	3.115657	0.895	0.129	0
V1	DCX	0	2.886347	0.992	0.305	0
V1	PCP4	0	2.814442	0.688	0.08	0
V1	LHX1	0	2.735357	0.755	0.027	0
V1	POU2F2	0	2.708875	0.716	0.08	0
V1	AC126175.2	0	2.503261	0.46	0.046	0
V1	ELAVL4	0	2.337274	0.882	0.202	0
V1	PCSK1N	6.19E-299	2.554213	0.854	0.244	1.52E-294
V1	TUBB3	1.69E-268	2.935331	1	0.896	4.16E-264
V1	MAP2	6.87E-267	2.477784	0.947	0.422	1.69E-262
V1	CDKN1C	7.01E-256	2.928421	0.994	0.588	1.73E-251
V1	PDLIM1	2.21E-250	3.121831	0.88	0.376	5.44E-246
V1	NEFL	6.73E-240	2.562243	0.854	0.322	1.66E-235
V1	NEFM	2.41E-222	2.435051	0.886	0.353	5.93E-218
V1	CXCR4	3.65E-115	2.400837	0.7	0.35	8.98E-111
V2a	VSX1	0	2.574677	0.416	0.005	0
V2a	ONECUT1	4.35E-297	2.222742	0.752	0.07	1.07E-292
V2a	SCG3	5.74E-162	2.107301	0.748	0.13	1.41E-157
V2a	INSM1	1.96E-158	2.137417	0.49	0.054	4.83E-154
V2a	ELAVL2	3.02E-143	2.090553	0.871	0.195	7.43E-139
V2a	PPP1R17	6.43E-141	2.677767	0.733	0.143	1.58E-136
V2a	ONECUT2	6.87E-126	2.736048	0.634	0.117	1.69E-121
V2a	TAGLN3	1.12E-94	2.480284	0.95	0.386	2.77E-90
V2a	TUBB3	1.58E-93	2.97471	1	0.897	3.89E-89

V2a	RPRM	3.13E-88	2.091184	0.911	0.359	7.71E-84
V2a	NRN1	6.16E-85	2.794807	0.292	0.036	1.52E-80
V2a	STMN1	5.32E-70	2.089685	1	0.929	1.31E-65
V2a	CRABP1	2.04E-69	3.113121	0.891	0.437	5.03E-65
V2a	MT1F	1.22E-68	2.451343	0.252	0.033	3.01E-64
V2a	STMN2	1.45E-66	2.953182	0.842	0.397	3.56E-62
V2a	HES6	5.43E-34	2.168652	0.832	0.586	1.34E-29
V2a	CKB	6.75E-33	2.159529	1	0.937	1.66E-28
V2a	IGFBP5	9.13E-28	3.234623	0.619	0.323	2.25E-23
V2a	MT1X	4.22E-20	4.580621	0.47	0.226	1.04E-15
V2a	SNCG	5.87E-19	2.228566	0.748	0.503	1.44E-14
V2b	MALAT1	8.66E-283	3.319681	1	0.983	2.13E-278
V2b	GRIA2	2.51E-267	3.21867	0.321	0.034	6.18E-263
V2b	WSB1	5.18E-215	3.262479	0.932	0.815	1.27E-210
V2b	MIAT	1.54E-187	3.727257	0.823	0.454	3.79E-183
V2b	KCNQ1OT1	1.81E-180	3.180039	0.907	0.755	4.46E-176
V2b	ONECUT1	1.26E-140	3.076196	0.344	0.07	3.10E-136
V2b	LINC00599	9.70E-140	3.144644	0.28	0.047	2.39E-135
V2b	NEAT1	3.32E-133	3.655311	0.784	0.597	8.17E-129
V2b	KIF5C	9.27E-112	3.662832	0.747	0.627	2.28E-107
V2b	NOVA1	3.73E-104	3.950624	0.667	0.432	9.18E-100
V2b	SRRM4	3.63E-99	3.176801	0.405	0.131	8.94E-95
V2b	POU2F2	4.31E-92	2.929532	0.323	0.086	1.06E-87
V2b	SYP	3.19E-88	3.026491	0.323	0.091	7.84E-84
V2b	AC006115.2	2.25E-69	3.509411	0.582	0.403	5.53E-65
V2b	RMST	1.35E-60	3.43254	0.541	0.355	3.32E-56
V2b	STARD4-AS1	5.59E-50	3.188817	0.469	0.292	1.38E-45
V2b	ONECUT2	9.47E-50	2.938687	0.302	0.118	2.33E-45
V2b	PCDH9	5.43E-46	3.068879	0.323	0.143	1.34E-41
V2b	TSHZ2	9.53E-39	3.393332	0.368	0.202	2.35E-34
V2b	NRXN1	1.29E-32	3.114135	0.5	0.431	3.18E-28
early mesoderm	RSPO3	0	2.322555	0.426	0.018	0
early mesoderm	DKK1	0	2.313347	0.429	0.009	0
early mesoderm	MSGN1	0	2.25307	0.462	0.003	0

early mesoderm	APLNR	0	1.927947	0.472	0.027	0
early mesoderm	FOXC2	0	1.907757	0.429	0.009	0
early mesoderm	HAPLN1	0	1.75466	0.371	0.015	0
early mesoderm	FOXC1	0	1.689381	0.534	0.004	0
early mesoderm	HAS2	0	1.62605	0.576	0.076	0
early mesoderm	COLEC12	0	1.598288	0.389	0.028	0
early mesoderm	CITED1	2.43E-249	2.49826	0.518	0.096	5.99E-245
early mesoderm	HES7	5.71E-226	1.530317	0.451	0.076	1.41E-221
early mesoderm	LEF1	5.33E-178	1.551185	0.683	0.213	1.31E-173
early mesoderm	COL1A2	7.39E-166	1.556654	0.739	0.26	1.82E-161
early mesoderm	PCOLCE	7.09E-153	1.546548	0.424	0.094	1.75E-148
early mesoderm	KRT19	1.61E-152	2.546227	0.65	0.215	3.95E-148
early mesoderm	ITM2A	2.81E-142	1.792342	0.516	0.149	6.91E-138
early mesoderm	MEST	3.38E-141	2.751874	0.954	0.715	8.32E-137
early mesoderm	SMC6	1.62E-119	1.906092	0.758	0.374	4.00E-115
early mesoderm	FABP5	3.81E-99	2.00874	0.971	0.83	9.39E-95
early mesoderm	NTS	2.31E-56	2.085785	0.317	0.108	5.69E-52

Supplementary data 3. List of differentially expressed genes in the neural progenitor clusters (Figure 3F related)

c1	c2	c3	c4	c5	c6
PLAAT3	MYH10	DKC1	KCNQ1OT1	SNHG5	APEX1
ANXA2	NRXN1	STMN1	U2SURP	ATP1A2	SNHG3
ARL4A	RPS4X	PGK1	NOP10	VAT1L	NDP
HOXC6	BCAR3	LMX1A	KRT19	ESRG	ADCY2
SEMA3E	SLC1A2	HNRNPH3	NTS	TNFRSF11B	HLA-C
DPYSL3	LINC00102	SMC3	KRT18	FOXB1	H2AFY
RPS8	DLK1	USP7	S100A11	PCDH9	NR2F2-AS1
RPLP0	FST	THRAP3	ZIC5	PARP1	CCDC140
RTN4	NKAIN4	ACSL4	SEMA3A	FAM136A	PAX3
ID3	MAPK12	PTCH1	BST2	YWHAZ	AP1S2
FGF13	FRZB	PDHB	CDX4	STK26	FAM110B
RPL28	TMEM64	LRAT	PSMA3	LARS	AC004540.2
POU3F4	PTX3	XPO1	PTMA	FOXH1	NRG1
CSPG5	LGR5	VRK1	HOXB7	SALL3	APCDD1
TMSB4X	F2R	DIAPH3	YBX1	SCGB3A2	PSMB1
TFDP2	FGFRL1	DCP2	PRICKLE1	STMN2	NEDD9
ERBB4	CD99	PCBP1	HNRNPD	SLC7A8	WNT4
AL139246.5	RGS20	CYCS	ACLY	TNS3	TPPP3
AC023794.3	TMEM120A	JARID2	HMGN1	MGST1	HHIP-AS1
HOXD3	TMEM132B	DNMT3B	CNTNAP2	DHX9	DACH2
TMSB15A	AL359546.1	EXOSC8	AC007906.2	BLM	VAT1
EDNRB	FAP	H3F3B	GREB1L	HIST1H3H	ADAMTS18
RPS12	MTTP	LMO4	UBE2K	FBXO5	CLEC3A
TTC3	LINC01198	B3GNT2	DENR	POLR3K	ZIC4
RPL5	HS3ST3A1	UCHL1	EIF2S2	SIVA1	HHIP
RPS13	SLC27A3	AL031058.1	ACTR2	RAD54L	TENT5A
MAPK10	RGMA	SPRY2	CCT4	LIG1	AC092958.1
RPS3	CDH7	LYAR	YBX3	WDHD1	GRID2
HOXC-AS1	FGFR3	SALL4	LIN28A	HIST1H2AK	SCTR
CXCR4	CELSR2	CD9	SLC39A8	HIST1H2BF	IGFBPL1
AP000781.1	FGFBP3	P4HA1	PFN1	HIST1H2AL	LYPD1
AC011773.	MYO10	HOXB6	PKIB	HSPD1	RPEL1

1					
EFNB2	SNCAIP	GNRH2	SSU72	ATP5MC3	TSC22D3
AL359091.1	SFRP1	TSSK1B	PTK7	NAA50	CALCB
ZFHX4	TCF7L1	AC012181.2	FGFR1	EIF2S1	C3orf80
MDK	LMCD1	AK4	RANBP1	TPM2	SNCG
ZNF503-AS2	DBX2	TCERG1L	TJP2	PABPC4	HTRA1
MYL6B	NAV3	CRYM	RAB3B	BCCIP	ADGRG1
DACH1	PRDM8	SPAG4	CLDN6	UCHL3	CCDC151
HNRNPK	WDR86	CYP1A1	GTF3C1	SF3A3	SYCE1L
MXD4	VEPH1	AC069431.1	BCLAF1	PSMG1	ATOH1
RND2	HS3ST3B1	RSPO3	LYN	CNKSR3	CLEC18A
AL583805.2	FAM181B	PKM	TSTD1	SLC25A5	HES6
CD24	SYT1	HLA-B	CACYBP	RPA3	RAB40B
COMMD6	KCNH8	BNIP3L	PCBP2	HAUS1	PFN2
PLPPR3	CLRN1	LINC00632	SAMD1	ETF1	IFITM3
E2F3	WNT7A	DDIT4	PA2G4	TPM3	RBP1
DHX36	GFRA1	HOXA6	S100A4	MSH2	GAPDH
PRTG	AC004543.1	CCDC80	NPM3	MCM8	RHOB
CLU	LINC01965	CITED2	EPHA4	USP1	HOXA9
CRABP1	VCAM1	SOX2	KRT8	HAT1	IGFBP2
FXYD6	MOXD1	GLIS3	CDX2	CENPX	TSHZ1
HES4	SOX21	NOL7	SERBP1	FKBP5	LINGO1
NCALD	PBX3	YWHAH	DMD	PPAT	ITM2C
ENPP2	DPYSL5	SMC2	GDF3	FGF17	MRPL3
TLE5	FHL1	UBE2T	SLC4A11	VASH2	GJA1
FJX1	SEPTIN11	C21orf58	ZIC2	APLP2	PIK3R1
SOX5	MCRIP1	MELK	LY6G6D	CDK2AP1	RPS6
KIF21A	FOXP4	IGFBP7	HOXC4	FTH1	RPL41
NES	SMIM14	GSPT1	ASXL3	NPM1	DKK3
CAMK2N1	CKB	SLC16A3	HOXA5	GNL3	RPL9
CFL1	FIS1	BNIP3	TFAM	XRN2	RPS27
IGDCC3	ZEB1	AL033527.4	MIF	RIF1	DCLK2
TSEN34	PLEKHO1	OR52N1	BMP4	DERA	EEF2
HS6ST1	DCTN2	SMC4	TPI1	EIF4EBP1	PCNA
MAGED2	IRX2	SALL1	FAM162A	TOPBP1	UHRF1
PLEKHA5	CRNDE	TCERG1	LIMCH1	GCSH	MCM10
METRN	CMTM3	HNRNPR	MAL	HSP90AB	HNRNPF

				1	
H3F3A	TSPAN13	HOXC8	ENO1	SLIRP	AC020661.3
HOXC9	LMO3	MXI1	LDHA	NDUFB1	MT-ND4
TMEM47	POU3F3	ARHGAP11A	ATP6AP1L	VDAC1	CDC25A
PPDPF	OPRK1	ANLN	NEFL	GLO1	ADI1
VAMP2	NDNF	NCAPG	DCUN1D5	RPL38	MT-ND2
DTX4	PALLD	HJURP	KRR1	CCT6A	NBDY
RPSA	LAMP5	RTKN2	SMIM3	SRSF7	DTL
RPL29	SLC12A2	CDKN2D	PSIP1	OAT	MCM5
PCSK1N	NRARP	CCNG2	PHLDB2	RASL10B	TIPIN
MEIS2	PROX1	EIF5	MRPS15	ATP6V1D	MSH6
RPS19	SOX2-OT	FOXM1	SNRPB	KMT2A	BEX3
RPL3	SOX3	NCAPH	WNT5A	PRKDC	SLBP
RPL10A	PANTR1	SPC25	HOXA-AS3	AC068587.4	WDR76
EEF1G	PCDH17	ZFP36L1	PABPC1	SNHG14	FAM111B
GPM6B	DIXDC1	FDFT1	L1TD1	IGF2BP1	UNG
TPT1	NAV2-AS4	EIF1	POLR3G	DNMT1	CASP8AP2
SRGAP3	CFAP299	PRRC2C	DUSP6	RFC1	RPS2
SH3BGRL	FZD7	TUBB2A	IL17RD	RPS17	RPL35A
C12orf57	FAT4	HSPH1	MRPL14	FXYD5	NACA
RACK1	SFRP2	UBC	FDPS	EPCAM	RPL7
RPL18A	PAX6	PSMD7	ALDH1A2	HNRNPU	RPS24
MIR99AHG	S1PR1	CENPW	DES	ARG2	EIF4A1
EEF1A1	COL5A2	SRSF3	LYPD6B	PPIA	ZIC3
RPS5	PARD3B	AMD1	PERP	SSRP1	RCN1
RPL18	GLUL	HNRNPA2B1	PSMB5	HSPB11	HNRNPM
WBP1	MAP2K5	AL512380.2	TLE4	RGS16	TSSK6
PRDM16	CBARP	RAN	PAK1IP1	PAICS	COX6C
MYC	CREB5	AL353747.4	RPS19BP1	FABP5	DANCR
SLC25A6	MSMO1	SACS	ANP32A	SPINT2	ZFP42
WFIKKN1	MSI1	CDCA5	TGS1	TCOF1	ZRANB2
COTL1	NRIP3	CALM1	YWHAB	SEPTIN9	CDCA7
UBE2E3	CAV1		H2AFZ	NUDT21	WNT7B
HOMER3	FILIP1		GPATCH4	SKP2	PDE1A
EIF3F	ANOS1		RRP1B	LINC01896	FEZ1
NTRK2	AUTS2		RNF24	NCL	RPL14
PTN	LGR4		SRRM2	SUPT16H	E2F1
BEX4	SHC2		CDK6	TYMS	PAQR4

RIPOR2	PALM		PTGES3	PCLAF	MT-CYB
PBX1	FZD2		INSIG2	DIAPH1	PHKA2-AS1
IFI27L2	SSX2IP		ENO2	STAG2	MT-ATP6
RPL13	TSC22D1		GPI	SRSF2	MT-CO2
RPS10	CDKN1C		PGAM1	CEBPZ	MT-CO1
RPS9	LRIG1		NEFM	DMKN	BASP1
HOXD8	DAAM1		ITGB5	BOLA3	MT-ND3
EMID1	PLCH1		SPRY1	GTF3A	AC136628.4
DLL1	ATF7IP		OLFM3	TNNT1	RPS15
ODC1	ITGA6		EOGT	FZD10	RPS25
TAF7	AC093388.1		SLC2A3	LSM6	RPL19
RFX4	TTC29		NAMPT	PODXL	RPL34
PRDX2	SLC7A11		BAX	IQGAP1	RPS7
CCDC160	NRXN3		REXO2	TPM4	RPL21
ARL4C	PALM2-AKAP2		TCP1	LRPPRC	RPS18
MEIS1	CSRNP3		WNT5B	ERVH48-1	RPLP1
SCUBE2	TUBA1A		UBE2L3	URAD	RPL32
PDCD4	EEF1D		SLC7A5	PRDX4	RPL17
MXRA8	MAP2K2		CCT5	TXNDC17	RPL11
BOC	MARCKS		CCT2	HOXB8	GAS5
PTMS	ARMCX3		SYNCRIP	CYYR1	ONECUT2
NOVA2	VPS28		MPP6	SNRPD1	LINC01780
LINC02381	BTG1		PFKP	SNRPG	NKD1
GABARAP	IRX5		NEAT1	TARS	MAP2
MEIS3	POU3F2		HOXB5	HPRT1	VCAN
UBXN1	BORCS7		H1F0	HNRNPC	RPL7A
TRAPPC1	PAK3		TRA2B	SERPINH1	RPL13A
REPIN1	CCN2		HNRNPAB	NQO1	RPL12
TLE1	PNRC1		ATF4	LEF1	RPS15A
CIRBP	NR2F1		ACTN1	CDX1	RPL30
	NR2F1-AS1		PSMD1	GNL3L	RPS28
	PHPT1		C3orf52	HSPA4	RPS16
	TFG		CGNL1	CDA	EVL
	ACADVL		NTM	DSP	ID2
	TOX3		ETV4	ACAT2	CNIH2
	ALKBH7		MYL12A	TIMP1	CST3
	MARCKSL1		MRPL13	APOC1	LIX1
	MEF2C		ETV5	SNRPN	C9orf16
	AP1S1		SRSF11	TXN	AC023794.

					1
	ACTG1		CDCA4	SUB1	C12orf76
	GPC3		CEP152	G3BP2	GLI3
	HOXA7		LHX1-DT	EIF1AX	TOX
	ISYNA1		SMC1A	HSPE1	SYBU
	BMP7		FANCI	UGT8	CTNND2
	APLP1		ESCO2	ARPC1B	KLHDC8B
	DBP		LSM3	TMEM14B	LRP2
	NPPC		SRPK1	GGCT	HOXC5
	FRMD4B		NR6A1	SELENOW	BMF
	HES5		ITM2A	CDK4	HOTAIRM1
	VIM		TRIM71	SLC16A2	QPRT
	PRSS23		NIPA2	IPO5	MIAT
	PHYHIPL		MND1	OAZ1	MAP6
	PDLIM5		ZWINT	DBI	MIR100HG
	NOTCH3		SYNE2	DSG2	REC8
	CRYGD		SMS	GSTO1	LINC00461
	FAT3		RBPMS	SPON1	HOXC-AS2
	PLP1		RRS1	ATP5MD	SEPTIN6
	ROBO2		ZFP36L2	AP2S1	TMEM121
	CELF2		RNPS1	PRDX3	FABP7
	QKI		SRSF4	BTG3	GABPB1-AS1
	MAPK11		CBX5	RAB34	SRP14
	TSPAN18		SLC3A2	PRPF40A	C1QTNF4
	ADAMTS6		EIF4G1	NME1	RHBDL3
	JAM2		CNMD	DNAJC8	ELOB
	TGFB2		VCL	NAE1	OTOGL
	KLHDC8A		RPA1	C1QBP	AC012409.2
	CPE		DDX46	DPM1	ANKRD66
	ID4		GBX2	SNRPA1	AC004988.1
	CXXC4		FAM153CP	UQCRH	EID1
	LFNG		HSPA9	CLTC	HNRNPA1
	FAM89A		XRCC5	PRDX1	MATN1
	TCF25		CHD4	NOP58	EPHA3
	TTYH1		TOMM40	APOE	MT-ND1
	ZFHX3		HIST1H3D	MMP2	EIF3E
	ZNF219		HIST1H2BC	NQO2	CCND1
	POR		HIST1H2AB	EEF1E1	MT-CO3

	PLTP		HIST1H2AG	HSP90AA 1	ZFAS1
	RPRM		HIST1H2AM	PSMA2	IGFBP5
	MIR124-2HG		HIST1H2AH	VAMP8	RPL4
	IRX3		HIST1H1B	ARPC3	RPL6
	ATP5MC2		HIST2H4B	PDIA6	RPS11
	ZNF428		HIST1H3A	NRP2	RPS14
	IRX1		HIST1H3B	ATP5MG	RPS3A
	HMGN3		HIST1H1E	CTSC	
	ITGA2		HIST1H1C	PPP1R14B	
	CRB2		HIST2H2BF	GSTP1	
	SCD5		HIST1H2BI	MICOS10	
	ITGB8		HIST1H4H	ALPL	
	FZD10-AS1		HIST2H2AA4	NME2	
	FAM181A		HIST1H2BO	TMA7	
	MYL6		HIST4H4	SET	
	KCNE5		IER2	SNRPF	
	LINC01414		HIST1H1A	AC104461.1	
	NOP53		HIST1H2BM	COX5B	
	ZBTB16		RRM2	SRM	
	VWA1		HIST1H2AC	ARPC2	
	SOX1		BAZ1B	PSMA4	
	LRRN1		BRCA2	JUNB	
	TUBB3		AFG3L2	HIST1H4E	
	PLXNA2		SUMO2	HIST1H3F	
	RPL10		GNG4	HIST1H3J	
	EIF3L		MTFP1	POLA1	
	LTBP4		MLLT3	HIST1H4I	
	GNAI2		SEMA6A	HIST1H2AE	
	SNHG29		TGFBI	HIST1H3I	
	NR2F2		RARG	RRM1	
	GSTM3		RBMS1	HIST1H4D	
	PCDH8		SPOCK3	HIST1H2BE	
	DDAH2		ARID3A	HIST1H1D	
	PGLS		ALYREF	KIF1A	
	GAS1		FGF2	POU5F1	
	UQCRFS1		PHLDA2	PLS3	
	PHF6		ZC3H18	VRTN	
	CD44		MLEC	SP5	
	ILF2		DDX21	SH3KBP1	

	CRIM1-DT	GSN	MIR302CHG
	POLR3GL	CTBP2	AC009654.1
	PRMT1	DLG1	CXCL12
	HOXD4	TUBA4A	GMNN
	GPX4	SCD	SLF2
	SIGIRR	UGCG	SCG3
	FAM229B	CALU	ADAMTS19
	TCEA2	PHGDH	NAP1L1
	CALM3	HNRNPA3	CHAF1A
	CCNI	NKX1-2	RMI2
	ZKSCAN1	RCC2	FANCA
	LIX1L	EIF3A	CDC45
	MAF1	MTHFD2	PRR14L
	RMST	IARS	GART
	GTPBP6	DHCR24	ANXA5
	WDR13	PTPRZ1	DHFR
	MID1	NOP56	SEMA5A
	TUBB2B	RAD51AP1	RPL27
	CEP70	DEK	CENPU
	AC026401.3	RANBP2	DSCC1
	SS18	HIST1H4C	SLC20A1
	RBMX	HIST2H2AC	BRCA1
	UBTF	HIST1H2AD	AKAP12
	MAP1B	PPM1G	PCAT14
	FGFR2	FOS	KLHL4
	PAFAH1B3	EGR1	MBNL3
	CADM1	RAB5IF	EXO1
	FAM89B	SRRM1	ICMT
	DCTN3	MYBL2	E2F7
	SPA17	CELF1	ATAD5
	PUF60	HIST1H2BH	OLFM1
	SOX11	HIST1H3G	ERP29
	ACBD7	NSD3	FAU
	ACTB	CENPK	DDR1
	DYNLRB1	KPNB1	WNT3
	RNF5	EEF1B2	RPL36
	UBB	ACYP1	RSPO1
	MLF2	ASF1B	ANKRD9
	AC004882.3	CCDC144NL-AS1	NCKAP5

	LGALS1		CUZD1	ATP5F1C	
	RHOU		JUND	LINC01887	
	HSP90B1			RAD51	
	ZC3H15			TRABD2A	
	SESN3			RPL31	
	MEGF10			RPL15	
	WDR54			TIMM8A	
	LARP7			SLC1A5	
	DNAJA1			GABRB3	
	GABARAPL2			MTRNR2L8	
	NARF			PUM3	
	BSCL2			SMARCA5	
	TXNIP			SOCS2	
	NDUFAF3			APELA	
	KDM5B			CD63	
	HMG20B			RPL37	
	TUBA1B			RPL27A	
	HNRNPA0			PGRMC1	
	PTTG1			RFC2	
	ZFAND5			MCM7	
	TXNDC12			RPL35	
	SFPQ			CDC6	
	CCDC167			MCM6	
	MORF4L2			RPL23	
	H1FX			CCNE1	
	GIHCG			MCM2	
	SOX4			POLD3	
	H2AFV			CLSPN	
	PLIN3			PPIF	
	GOT1			DSTN	
	NUDCD2			PRPS2	
	SINHCAF			NDUFA4	
	PRRX2			ANP32B	
	ASCL1			COX7C	
	HSPA8			RPS29	
	CCAR1			RPLP2	
	FXR1			RPL36A	
	GSX2			RPL39	
	PRDM13			RPS21	
	HSD17B11			NEDD8	

	ZNF503			WLS	
	PNISR			ABRACL	
	SCLT1			ATP1B3	
	CCNB1			SEC61B	
	TROAP			NDUFB4	
	MYEF2			HDAC2	
	APOLD1			POMP	
	BIRC5			ABCE1	
	PLK1			LINC01315	
	KNSTRN			RTRAF	
	FZR1			NDUFAB1	
	LBR			CDC123	
	UBE2C			PFDN4	
	FAM110A			PSMA5	
	PPP1R35			TOMM7	
	TRIOBP			PDIA3	
	SKA2			SPARCL1	
	PARPBP			LDHB	
	RNF26			ATP5ME	
	BORA			CSTF3	
	RAD21			ATP5PF	
	MZT1			NDUFB2	
	ARHGEF39			HINT1	
	HMMR			GLRX3	
	ODF2			LAGE3	
	DDX39A			RPS26	
	PSRC1			NDUFS5	
	ASPM			PSMA7	
	SLC4A8			ATP5MF	
	VPS35			YWHAE	
	SELENOK			SSBP1	
	HYLS1			ENY2	
	BUB3			UTP20	
	C2orf69			COX7B	
	CALM2			BZW1	
	AC013643.2			TMSB10	
	CDKN1B			AXIN2	
	MN1			FKBP3	
	NBAS			PDCD5	
	EDRF1-AS1			SUMO1	

	PRPSAP1			RPL24	
	TNFAIP8L1			HDDC2	
	EIF1B			SEM1	
	KATNA1			SNRPE	
	MEX3A			FTL	
	SUN2			SCG5	
	LMNB1			RARRES2	
	HDGF			HOXA3	
	AL162171.1			CA4	
	MALAT1			AC103702.2	
	TUBA1C			BACE2	
	GPSM2			CSRP2	
	ARL6IP1			RPL22	
	CCNB2			RPS27A	
	CDC20			CHAF1B	
	HMGB3			DCTPP1	
	HSPA1A			KNOP1	
	KPNA2			RFC4	
	NEK2			DONSON	
	HP1BP3			EXOSC9	
	NDE1			TEX15	
	TUBB4B			FEN1	
	PHF19			PEG10	
	TBCB			DUT	
	FZD3			RPS20	
	TMEM106C			SLIT2	
	PIAS1			CTNNAL1	
	P4HA2			MESD	
	HABP4			EBNA1BP2	
	JUN			PLSCR1	
	MRPL51			NOLC1	
	TOP1			TFDP1	
	AL158835.1			EIF5B	
	CLIC1			NASP	
	CDK5RAP2			RPL23A	
	MIS18BP1			RPL37A	
	TUBB			PNN	
	KIF11			MCM4	
	KLF6			RECQL	
	KIF5B			GINS2	

	DEPDC1			UQCRB	
	UACA			SELENOH	
	NDC80			PPIB	
	CDCA2			HELLS	
	DBF4			RBBP8	
	MKI67			MCM3	
	FAM83D			NETO2	
	KIF23			ATAD2	
	PIF1			RPL26	
	CDC27			RPS23	
	CIT			SLC16A1	
	H2AFX			SSB	
	NUSAP1			EIF4G2	
	FAM122B			S100A10	
	KIF22			SLC8A1	
	ARHGAP19			UNC5C	
	HMGB2			ISM1	
	PNRC2			LINC01933	
	BUB1B			LINC01116	
	KNL1			CRIP1	
	PRC1			COL14A1	
	SPAG5			CD82	
	CKAP2L			IGF2-AS	
	RACGAP1			RND3	
	FAM72D			NDUFA6	
	NUCKS1			KDELR1	
	RBM8A			DRAXIN	
	EMC9			HOXA4	
	TUBB6			FBXO2	
	DYNLL1			HOXB4	
	ANP32E			WNT3A	
	HMGB1			COL2A1	
	CHORDC1			PLAGL1	
	RHEB			CCND2	
	RHNO1			C4orf3	
	BRD8			CASZ1	
	KIFC1			HOXA10	
	AURKA			CDC42EP5	
	CKAP5			HOXC10	
	UBE2S			HOXD9	

	NCAPD2			IGF2	
	TPX2			HOXB-AS3	
	CKS2			CRABP2	
	CCDC18			MEST	
	KIF2C			HES1	
	CCNF			HOXB9	
	GTSE1			GDF10	
	CEP55			NEUROG2	
	CCNA2			RAB38	
	PRR11			OLIG3	
	DLGAP5			MYOF	
	CENPA			BX324167.1	
	CKS1B			EIF5A	
	KIAA0586			MSX2	
	INCENP			ZIC1	
	KIF14			AC078785.2	
	CDCA8			FKBP1A	
	AURKB			NME4	
	KIF20B			SEC61G	
	KIF20A			HOXB3	
	CENPE			ALDOC	
	KIF4A			BCL11A	
	ECT2			WNT1	
	BUB1			ID1	
	SAPCD2			CTHRC1	
	CDKN3			MSX1	
	CDCA3			AC103702.1	
	UBALD2			GREM1	
	JPT1			DHRS3	
	SGO2			LAPTM4A	
	DCAF7			MTPN	
	TRIM59			TPBG	
	KIF18A			ZNF775	
	RANGAP1				
	GAS2L3				
	REEP4				
	G2E3				
	CDC25C				
	NUF2				
	TTK				

	DEPDC1B				
	CENPF				
	DTYMK				
	KMT5A				
	CDK1				
	MXD3				
	SGO1				
	HMGN2				
	PIMREG				
	CKAP2				
	TACC3				
	MAD2L1				
	PBK				
	SP8				
	TOP2A				
	WSB1				
	KIF15				
	CIP2A				
	TMPO				
	SLC38A1				
	ARF1				
	NECTIN3				
	AL118516.1				
